# Comparative genomics highlights the importance of drug efflux transporters during evolution of mycoparasitism in *Clonostachys* subgenus *Bionectria* (Fungi, Ascomycota, Hypocreales)

**DOI:** 10.1111/eva.13134

**Published:** 2020-09-28

**Authors:** Martin Broberg, Mukesh Dubey, Mudassir Iqbal, Mikael Gudmundssson, Katarina Ihrmark, Hans‐Josef Schroers, Dan Funck Jensen, Mikael Brandström Durling, Magnus Karlsson

**Affiliations:** ^1^ Department of Forest Mycology and Plant Pathology Swedish University of Agricultural Sciences Uppsala Sweden; ^2^ Department of Molecular Sciences Swedish University of Agricultural Sciences Uppsala Sweden; ^3^ Agricultural Institute of Slovenia Ljubljana Slovenia

**Keywords:** antagonism, biological control, *Clonostachys*, membrane transporter, mycoparasitism, xenobiotics

## Abstract

Various strains of the mycoparasitic fungal species *Clonostachys rosea* are used commercially as biological control agents for the control of fungal plant diseases in agricultural crop production. Further improvements of the use and efficacy of *C. rosea* in biocontrol require a mechanistic understanding of the factors that determines the outcome of the interaction between *C. rosea* and plant pathogenic fungi. Here, we determined the genome sequences of 11 *Clonostachys* strains, representing five species in *Clonostachys* subgenus *Bionectria*, and performed a comparative genomic analysis with the aim to identify gene families evolving under selection for gene gains or losses. Several gene families predicted to encode proteins involved in biosynthesis of secondary metabolites, including polyketide synthases, nonribosomal peptide syntethases and cytochrome P450s, evolved under selection for gene gains (*p* ≤ .05) in the *Bionectria* subgenus lineage. This was accompanied with gene copy number increases (*p* ≤ .05) in ATP‐binding cassette (ABC) transporters and major facilitator superfamily (MFS) transporters predicted to contribute to drug efflux. Most *Clonostachys* species were also characterized by high numbers of auxiliary activity (AA) family 9 lytic polysaccharide monooxygenases, AA3 glucose–methanol–choline oxidoreductases and additional carbohydrate‐active enzyme gene families with putative activity (or binding) towards xylan and rhamnose/pectin substrates. Particular features of the *C. rosea* genome included expansions (*p* ≤ .05) of the ABC‐B4 multidrug resistance transporters, the ABC‐C5 multidrug resistance‐related transporters and the 2.A.1.3 drug:H + antiporter‐2 MFS drug resistance transporters. The ABC‐G1 pleiotropic drug resistance transporter gene *abcG6* in *C. rosea* was induced (*p* ≤ .009) by exposure to the antifungal *Fusarium* mycotoxin zearalenone (1121‐fold) and various fungicides. Deletion of *abcG6* resulted in mutants with reduced (*p* < .001) growth rates on media containing the fungicides boscalid, fenhexamid and iprodione. Our results emphasize the role of biosynthesis of, and protection against, secondary metabolites in *Clonostachys* subgenus *Bionectria*.

## 1 INTRODUCTION

Parasitic interactions of a fungus on other living fungi involving penetration of host or prey hyphae are referred to as mycoparasitism (Barnett, 1963). Mycoparasitic relationships display a range from biotrophic, where nutrients are acquired from living host cells, to necrotrophic, where the fungal prey is killed and subsequently consumed by the mycoparasite (Barnett, 1963; Karlsson et al., 2018). Certain mycoparasitic species are used as biological control agents (BCAs) against plant pathogenic fungi in agricultural production settings (Jensen et al., 2016), and their use has been demonstrated to be a more sustainable and less hazardous alternative to chemical pesticides or antimicrobial compounds in agriculture (Harman et al., 2004; Vardhan et al., 2013). Knowledge‐based improvement of the application and use of fungal mycoparasites as BCAs requires an understanding of the mechanistic basis for the interaction between parasite and prey.

Several of the commercially most successful biocontrol products are based on fungi from the order Hypocreales, especially the genera *Clonostachys* and *Trichoderma*. As ubiquitous filamentous fungi, certain species of *Clonostachys* and *Trichoderma* share an ecological role as generalists, being able to feed on multiple nutrient sources including live and dead fungi (mycotrophy) and dead plant or animal material (polyphagy) (Karlsson et al., 2018). Representatives of both genera are aggressive necrotrophic mycoparasites with a broad host range, including both alloparasitism (parasitism on unrelated hosts) as well as adelphoparasitism (parasitism on closely related hosts) (Druzhinina et al., 2011; Jensen et al., 2007). Phylogenetic studies have proposed that several lifestyle transitions have occurred within the Hypocreales (Spatafora et al., 2007) and the mycoparasitic lifestyle is no exception. *Clonostachys rosea*, *Trichoderma* spp. and *Tolypocladium ophioglossoides* are all mycoparasitic species from the hypocrealean Bionectriaceae, Hypocreaceae and Ophiocordycipitaceae families, respectively. These lifestyle transitions have had a significant impact on the evolutionary history of the involved species and on their genome content (Karlsson et al., 2015; Kubicek et al., 2011, 2019; Quandt et al., 2015).

Certain strains of *C. rosea* (Link) Schroers, Samuels, K.A. Seifert & W. Gams [#461067] have been demonstrated to provide efficient control of many diseases caused by plant pathogenic fungi and even nematodes (Iqbal, et al., 2018; Knudsen et al., 1995; Sutton et al., 1997; Xue et al., 2009). Active ingredients of marketed products of this species may also be filed as *Gliocladium catenulatum* or *G. roseum*, which are considered synonyms of *C. rosea*. The extensive literature concerning the biocontrol properties of *C. rosea* is in sharp contrast to the lack of reports concerning the beneficial traits of other *Clonostachys* species. *Clonostachys chloroleuca* G.M. Moreira, L.M. Abreu, L.H. Pfenning & H.J. Schroers [#816994] strain 67‐1 (previously reported as *C. rosea*) can control sclerotinia stem rot on soybean caused by *Sclerotinia sclerotiorum* (Sun et al., 2017), while *C. byssicola* Schroers [#485119] is reported to control frosty pod rot caused by *Moniliophthora roreri*, black pod by *Phytophthora* spp. and rosellinia root rot in cocoa by *Rosellinia* spp. (Garcia, ten Hoopen, Kass, Garita, & Krauss, 2003; Krauss et al., 2006). This raises the question whether factors that enable *Clonostachys* to be exploited for biocontrol applications are strain, species or genus specific or whether different *Clonostachys* taxa can be distinguished from each other according to their beneficial traits.

*Clonostachys rosea* can attack and kill plant pathogenic fungi as a mycoparasite (Barnett & Lilly, 1962; Li et al., 2002; Yu & Sutton, 1997), an interaction that involves fungal cell wall‐degrading enzymes such as chitinases, glucanases and proteases (Chatterton & Punja, 2009; Li et al., 2006). Previous studies also demonstrate that *C. rosea* can produce antimicrobial compounds such as peptaibols, epipolysulfanyldioxopiperazines and polyketides (Dong et al., 2005; Fatema et al., 2018; Rodriguez et al., 2011; Zhai et al., 2016). Detoxification and efflux of the antifungal *Fusarium* spp. mycotoxin zearalenone (Utermark & Karlovsky, 2007) enables *C. rosea* to more efficiently antagonize *F. graminearum*, consequently resulting in better protection of wheat and barley against foot rot disease (Dubey et al., 2014a; Kosawang, et al., 2014). The ability of *C. rosea* to compete with other fungi for space and nutrients also contributes to its application as a BCA in crop production (Yu & Sutton, 1997). Plant growth promotion and induction of plant defence reactions by *C. rosea* is also reported (Lahlali & Peng, 2014; Mouekouba et al., 2014; Ravnskov et al., 2006; Roberti et al., 2008).

Based on a comprehensive comparative genome analysis of *C. rosea* strain IK726, the most distinguishing features are gene copy number expansions of gene families encoding ankyrin‐repeat proteins, ATP‐binding cassette (ABC) transporters, major facilitator superfamily (MFS) transporters, polyketide synthases (PKS), cytochrome P450 monooxygenases (CYP), auxiliary activity family 3 (AA3) glucose–methanol–choline oxidoreductases, AA9 lytic polysaccharide monooxygenases, polysaccharide lyase family 1 (PL1) pectin/pectate lyases and serine proteases, when compared to plant pathogenic *Fusarium* species and mycoparasitic *Trichoderma* species from the order Hypocreales (Iqbal, et al., 2018; Karlsson et al., 2015; Nygren et al., 2018). However, as the *C. rosea* strain IK726 genome is so far the only thoroughly analysed Bionectriaceae genome, it is difficult to assess whether these gene family expansions are characteristic for strain IK726, the species *C. rosea*, the genus *Clonostachys* or even the Bionectriaceae.

For *C. rosea*, efflux of antifungal compounds by ABC transporters ABCG5 and ABCG29 is reported as a factor reducing the plant adverse potential of plant pathogenic fungi (Dubey et al., 2014a, 2016), and for *T. atroviride* by TaABC2 (Ruocco et al., 2009). TaPDR2 from *T. atroviride* provides tolerance against the insect pesticide dichlorvos (Sun et al., 2019). ABC transporters typically contain two cytoplasmic nucleotide‐binding domains (NBD) and two membrane‐associated transmembrane domains (TMD) (Lamping et al., 2010). Several conserved motifs are present within the NBD, including Walker A (P‐loop), Q‐loop, ABC signature motif (C‐loop), pro‐loop, Walker B, D‐loop and switch region (H‐loop), together responsible for binding and hydrolysis of ATP to drive membrane transport (Lamping et al., 2010). Each TMD typically contain six transmembrane α‐helices. Sequence similarity and domain topology are used to divide ABC transporters into ten different groups (Kovalchuk & Driessen, 2010), where ABCG5 and ABCG29 from *C. rosea* and TaABC2 and TaPDR2 from *T. atroviride* are all classified as members in group G that accommodates the pleiotropic drug resistance efflux pumps (Kosawang et al., 2014; Ruocco et al., 2009; Sun et al., 2019).

In the current work, we performed an in‐depth comparative genomic analysis of *C. rosea* and several other *Clonostachys* species of subgenus *Bionectria*, including *C. byssicola*, *C. chloroleuca*, *C. rhizophaga* Schroers [#485120], *C. rosea*, *C. solani* (Harting) Schroers & W. Gams [#456098] and *Clonostachys* sp. Selected species have a shared common ancestor and similar morphological characters (Moreira et al., 2016; Schroers, 2001; this study). The approach allows thus to increase our understanding of the evolution of factors involved in mycoparasitism, plant beneficial traits and drug efflux in *Clonostachys* subgenus *Bionectria*.

## 2 MATERIALS AND METHODS

### 2.1 Strains, culture conditions and DNA extraction

Taxonomic authorities of species used in the current study are provided in Table S1. *Clonostachys* strains (Table 1) including *C. rosea* strain IK726, *F. graminearum* Schwabe [#200256] strain PH‐1 and *Botrytis cinerea* Persoon [#217312] strain B05.10 were revived from glycerol stocks stored at −80°C and maintained on potato dextrose agar (PDA; Oxoid, Cambridge, UK) Petri dishes at 25°C in darkness. Conidia were harvested from 2‐week‐old PDA Petri plates and counted using a haematocytometer as described previously (Fatema et al., 2018). For DNA extraction, *Clonostachys* strains were grown in 200 ml liquid Czapek‐Dox medium (Sigma‐Aldrich, Steinheim, Germany), Vogel´s minimal medium (Vogel, 1956) or malt extract (1.75%) with peptone (0.25%) medium at room temperature on a rotary shaker set to 120 rpm. Fungal biomass was harvested by filtration after 3–13 days, depending on growth rate, frozen in liquid nitrogen followed by freeze‐drying and storage at −80°C. High‐quality genomic DNA was extracted using CTAB/chloroform‐based protocol or the Qiagen‐tip 100 kit (Qiagen, Hilden, Germany) as described previously (Broberg et al., 2018).

**Table 1:** Genome statistics for the studied strains of different *Clonostachys* species

Genus	Species	Strain ID	Origin	Sequencing technology	Sequence alignment^a^ (%)	Genome size (Mbp)	N50^b^ (kbp)	Gene count
*Clonostachys*	*byssicola*	CBS 245.78	Brazil	Illumina HiseqX	45.6	54.9	890	18,541
*Clonostachys*	*byssicola*	CBS 115882	Argentina	Illumina HiseqX	43.2	57.2	464	n.a.
*Clonostachys*	*byssicola*	CBS 113336	South Africa	Illumina HiseqX	44.7	55.5	791	n.a.
*Clonostachys*	*byssicola*	CBS 552.84	Japan	Illumina HiseqX	41.9	57.4	704	n.a.
*Clonostachys*	*chloroleuca*	CBS 570.77	Egypt	Illumina HiseqX	52.6	61.1	633	19,658
*Clonostachys*	*chloroleuca*	CBS 100495	Australia	Illumina HiseqX	54.5	57.1	324	n.a.
*Clonostachys*	*chloroleuca*	CBS 227.48	USA	Illumina HiseqX	55.3	57.4	598	n.a.
*Clonostachys*	*rhizophaga*	CBS 906.72A	Chile	Illumina HiseqX	55.2	59.4	615	18,962
*Clonostachys*	*rhizophaga*	CBS 229.48	USA	Illumina HiseqX	56.4	55.8	1,404	n.a.
*Clonostachys*	*solani*	1703	Slovenia	PacBio RSII	‐	54.5	1,800	18,093
*Clonostachys*	sp.	CBS 192.96	Papua New Guinea	Illumina HiseqX	19.5	53.0	220	18,459

Abbreviation: n.a., ot annotated.

^a^ In comparison with the *C. rosea* strain IK726 reference genome (Broberg et al., 2018).

^b^ N50 values refer to scaffolds, except for strain 1703 where it refers to contigs.

Species identity of genome‐sequenced strains was confirmed also based on macro‐ and micromorphological characters, thus colony appearances and size and shape of conidia and conidiophore branching patterns (Moreira et al., 2016; Schroers, 2001; Schroers et al., 1999). Strains were grown on potato dextrose and oatmeal agar (BD Difco, Le Pont de Claix, France), and carrot potato agar (Gams et al., 1998) in 9‐cm Petri plates incubated at 25ºC for 7–14 days. Structures were mounted in water or lactic acid and looked at on a Zeiss Imager Z1 equipped with an Axiocam MRc5 camera and AxioVS40 software.

### 2.2 In vivo biocontrol assay

The ability of *Clonostachys* spp. to protect wheat (*Triticum aestivum*, winter wheat variety “Stava”) against fusarium foot rot disease, induced with *F. graminearum* strain PH‐1, was evaluated in a climate chamber bioassay in five biological replicates, where each replicate contained 12–15 plants, following the procedure as described previously (Dubey et al., 2014a; Knudsen et al., 1995). Seedlings were harvested 3 weeks postinoculation, and disease symptoms were scored based on a 0 to 4 scale as described previously (Dubey et al., 2014a; Knudsen et al., 1995).

### 2.3 In vitro assay of antagonism

In vitro antagonistic ability of *Clonostachys* spp. against *F. graminearum* was examined using a plate confrontation assay on PDA agar plates (Dubey et al., 2014a, 2016). A 5‐mm mycelial agar plug of *Clonostachys* spp. was inoculated at the edge of 9‐cm Petri plate. After seven days of incubation at 25°C in darkness, an agar plug of *F. graminearum* was inoculated at equal distance to the opposite edge of the plate. Mycelial growth was measured in five biological replicates three days postinoculation. Controls were based on plates inoculated only with *F. graminearum*.

### 2.4 Genome sequencing, assembly and annotation

Base coverage of the *C. solani* strain 1703 genome was generated using PacBio RSII Technology with an insert length of 20 kbp using standard library preparation kits. The PacBio reads were subjected to error correction of the longest reads by subread filtering, mapping and de novo assembly of all reads into long polished contigs using HGAP ver. 3.0 and the SMRT pipeline (Chin et al., 2013; Roberts et al., 2013). Base coverage of additional *Clonostachys* genomes was generated using Illumina HiSeqX paired end sequencing with an insert length of 350 bp and read length of 150 bp using standard library preparation kits. Genomes were assembled using ABySS ver. 1.3.6 (Simpson et al., 2009). Bowtie2 ver. 2.2.4 was used to calculate the alignment between sequenced strain reads and the *C. rosea* genome (Langmead & Salzberg, 2012). Genomes were annotated using a MAKER‐based pipeline described previously (Karlsson et al., 2015). The assembled genomes of *C. rosea* strain YKD0085 (Liu et al., 2016), *C. chloroleuca* strain 67‐1 (Sun et al., 2015a), *T. atroviride* strain IMI 206,040 (Kubicek et al., 2011), *T. reesei* strain QM6a (Martinez et al., 2008), *T. virens* strain Gv29‐8 (Kubicek et al., 2011), *F. graminearum* strain PH‐1 (Cuomo et al., 2007), *Neocosmospora* sp. strain 77‐13‐4 (=*Fusarium solani* MPVI) (Coleman et al., 2009), *F. verticilloides* strain 7,600 (Ma et al., 2010) and *Neurospora crassa* strain OR74A (Galagan et al., 2003) were re‐annotated using the same annotation pipeline as for the *Clonostachys* spp. genomes. The annotations from MAKER were used to classify the genome sequences into functional categories. To assess the presence of repeat‐induced point mutation (RIP) in repeat sequences, the composite RIP index as defined by Lewis et al. (Lewis et al., 2009) was calculated. RIP was calculated for the concatenated sequences of each repeat category, as defined by the MAKER repeat annotations.

### 2.5 Gene family prediction and annotation

The annotated proteomes of all strains were analysed with OrthoFinder ver. 1.1.8 (Emms & Kelly, 2015) to detect orthologous genes between species, and to construct groups of orthologous genes. Orthology clusters were annotated using a protein domain sequence coverage (Gene Ontology and InterPro categories) cut‐off of >75% for each cluster, aggregating the overall annotation using Kinfin (Laetsch & Blaxter, 2017). Details of the used commands are listed in Table S2.

Predicted proteins for all species were compared with transporter proteins in the Transporter Classification database (TCDB) (Saier et al., 2016) using an e‐value threshold of <10^–5^ as cut‐off and best‐hit to determine annotation to different transporter categories. ABC transporters were further classified according to the Kovalchuk and Driessen classification system (Kovalchuk & Driessen, 2010) as described previously (Karlsson et al., 2015; Nygren et al., 2018). All predicted proteins were also compared with carbohydrate‐active enzymes in the dbCAN CAZyme database (CAZyDB.07202017.fas) using an e‐value threshold of <10^–5^ as cut‐off and best‐hit to determine representation of the different CAZy categories (Lombard et al., 2014; Yin et al., 2012). We also performed a more stringent analysis using HMMER3 alignment of the dbCAN Hidden Markov Model (HMM) database, using an e‐value threshold of <10^–17^ and a coverage >0.45 (Finn et al., 2011). Clusters of secondary metabolite biosynthesis genes were identified using antiSMASH ver. 3.0 (Weber et al., 2015).

### 2.6 Phylogenetic analyses

Gene sequences of ATP citrate lyase (*acl1*), RNA polymerase II large subunit (*rpb1*), translation elongation factor 1‐α (*tef1*) and β‐tubulin (*tub*) (Table S3) of all *Clonostachys* strains and a reference set from Moreira et al. (2016) were aligned individually with MUSCLE (Edgar, 2004) and concatenated. Phylogenetic analyses were performed using maximum likelihood methods implemented in MEGA ver. 6 (Tamura et al., 2013) using all sites, and 1,000 bootstrap resamplings. The best substitution models were identified in MEGA ver. 6 and were GTR+G for *acl1*, TN93+G for *rpb1*, K2+I for *tef1*, K2+G for *tub* and TN93+G for the concatenated dataset. A species tree topology was generated by OrthoFinder ver. 1.1.8 (Emms & Kelly, 2015) according to standard programme settings, based on all predicted orthogroups. Gene sequences of *acl1*, *tef1* and *rpb1* were aligned individually with MUSCLE (Edgar, 2004), concatenated and used to calculate branch lengths in MEGA ver. 6 (Tamura et al., 2013). The split between *T. reesei* and *T. virens* at 16 million years ago (de Man et al., 2016) was used to calibrate the resulting species phylogeny.

Predicted ABC transporter protein sequences were aligned with MUSCLE (Edgar, 2004), and phylogenetic analysis was performed using maximum likelihood methods implemented in MEGA ver. 6 (Tamura et al., 2013). The LG (Le & Gascuel, 2008) +G+F amino acid substitution model was used for all sites. Statistical support for branches was assessed based on 500 bootstrap resamples.

### 2.7 Analysis of evolution of gene family composition

The generated species phylogeny and predicted gene families were used to test for a stochastic birth‐and‐death evolution of gene family size, to estimate gene family size in extinct species and to identify lineages with accelerated rates of gene gain or loss using the CAFE ver. 3.1 software (Han et al., 2013) and a *p*‐value cut‐off at ≤.05. Gene families were filtered for abundances, with the requirement of ≥2 species with ≥1 gene, and ≥1 species with ≥ 2 genes, as done previously (Nygren et al., 2018). Separate mutation rates (λ) were calculated for branches leading to *Clonostachys*, *Fusarium*, *Trichoderma* and *Neurospora*. Details of the used commands are listed in Table S2.

The software package NOTUNG ver. 2.9 (Darby et al., 2017) was used for reconciliation analysis of an ABC transporter phylogenetic tree and a *Clonostachys* species tree. The analysis was performed with default parameters, that is 1 for losses and 1.5 for duplications and with a 90% bootstrap cut‐off in order to collapse poorly supported topologies.

### 2.8 Analysis of molecular evolution and homology modelling of ABC transporters

Regions of low amino acid conservation in ABC transporter alignments were identified by reverse conservation analysis (RCA, [Lee, 2008]). In short, Rate4Site ver. 2.01 was used to calculate the degree of conservation (S score, high scores correspond to low degree of conservation) for each amino acid position using the empirical Bayesian method (Mayrose et al., 2004; Pupko et al., 2002). A sliding‐window average (*n* = 7) of normalized S scores (mean was 0 and standard deviation was 1) was plotted in Excel (Microsoft) (W mean score), and significant peaks were defined by values ≥1. Transmembrane helices were predicted using TMHMM ver. 2 (Krogh et al., 2001). Conserved protein modules and features were identified using the simple modular architecture research tool (SMART) (Letunic and Bork, 2017).

Homology models of ABCG6 were generated via the I‐TASSER server (Roy et al., 2010; Yang & Zhang, 2015; Zhang, 2008). The ABCG6 model was composed of an assembly of two protein chains, with an I‐TASSER confidence score (C‐score) of −1.90 and −1.14. Molecular images were made using PyMOL (The PyMOL Molecular Graphics System, Version 2.0, Schrödinger, LLC.).

### 2.9 Gene expression analysis

Gene expression analysis was carried out during dual culture PDA plate interactions of *C. rosea* with *B. cinerea* or *F. graminearum*, and in liquid Czapek‐Dox (CZ) medium amended with zearalenone (Sigma‐Aldrich, St. Louis, MO) and fungicides such as Apron XL (Syngenta, Basel, Switzerland; active ingredient mefenoxam), Amistar (Syngenta Basel, Switzerland; active ingredient azoxystrobin), Chipco green 75WG (Bayer, Leverkusen, Germany; active ingredient iprodione), Cantus (BASF Canada Inc., Mississauga, Canada; active ingredient boscalid) or Teldor (Bayer, Leverkusen, Germany; active ingredient fenhexamid) to a final concentration of 10 μg/ml (zearalenone), 2 μg/ml (mefenoxam), 7.5 μg/ml (azoxystrobin), 250 μg/ml (iprodione), 2000 μg/ml (boscalid) or 2,500 μg/ml (fenhexamid) following procedures described previously (Dubey et al., 2014a, 2016). For gene expression analysis during interactions, the growing front of *C. rosea* mycelium was harvested when the colonies of both species got into contact (Dubey et al., 2014a, 2016). Mycelium harvested at same stage from *C. rosea* confronted with itself was used as control treatment. For gene expression analysis in CZ medium, mycelium was harvested after 2 hr of exposure to the zearalenone or fungicides, washed several times in distilled water to remove traces of fungicides, frozen in liquid nitrogen and stored at −80°C. The experiment was performed in five biological replicates.

RNA extraction was done using the Qiagen RNeasy kit following the manufacturer's protocol (Qiagen, Hilden, Germany). After DNaseI (Fermentas, St. Leon‐Rot, Germany) treatment, RNA quantity and quality were determined using a 2,100 Bioanalyzer Instrument (Agilent Technologies, Santa Clara, CA). One microgram of total RNA was reverse transcribed (RT) in a total volume of 20 μl using the iScript cDNA synthesis kit (Bio‐Rad, Hercules, CA). Transcript levels of *abcG6* were quantified by RT‐qPCR using the SsoFast EvaGreen Supermix (Bio‐Rad, Hercules, CA) and primer pair abcG6 F/abcG6 R (Table 2) in an iQ5 qPCR System (Bio‐Rad, Hercules, CA) as described previously (Tzelepis et al., 2012, 2015). Relative expression levels for target genes in relation to *tub*, shown previously to be constitutively expressed (Dubey et al., 2014a, 2016; Kamou et al., 2016; Tzelepis et al., 2015), were calculated from the Ct values using the 2^‐∆∆Ct^ method (Livak & Schmittgen, 2001). Gene expression analysis was carried out using five biological replicates, each based on two technical replicates.

**Table 2:** List of primers used in this study

Primer name	Sequence (5´→3´)
abcG6 F	TTCGGACTTGGGTTTGCCTTTAT
abcG6 R	TTTGACATGGCAAGTTCCGTTCTA
β‐tubulin F	GGTCAGTGCGGTAACCAAAT
β‐tubulin R	ACAGCGCGAGGAACATACTT
abcG6‐ups F^a^	ggggacaactttgtatagaaaagttgTTCCTTCTCGAGATGCTGATTTGA
abcG6‐ups R^a^	ggggactgcttttttgtacaaacttgAGAGAATTGGCGGGCGAGACT
abcG6‐ds F^a^	ggggacagctttcttgtacaaagtggCCAAAGGGCAGGAAGAAGCAGTA
abcG6‐ds R^a^	ggggacaactttgtataataaagttgCTGCCATCGGAGGTGCTTGTT
abcG6 ko F	CCTCCTTCGTTTTCGGTGTGGT
abcG6 ko R	GTCCCGCACGAAGCTCCTATCT

^a^ attB and attBr sequences for multisite gateway BP recombination are presented in lowercase.

### 2.10 Construction of gene deletion vector, transformation and mutant validation

Phusion DNA polymerase (Finnzymes, Vantaa, Finland) was used for PCR amplification of ~1 kb 5′ flank and 3′ flank regions of the *abcG6* gene from genomic DNA of the wild‐type *C*. *rosea* strain IK726 using primer pairs abcG6‐ups F/abcG6‐ups R and abcG6‐ds F/abcG6‐ds R, respectively (Table 2). Gateway entry clones of the purified 5′‐flank and 3′‐flank PCR fragments were generated as described by the manufacturer (Invitrogen, Carlsbad, CA). The gateway entry clone for hygromycin (*hph*) gene constructed previously (Dubey et al., 2012, 2013; Dubey, et al., 2013) was used. The gateway LR recombination reaction was performed using entry plasmid of respective fragments and destination vector pPm43GW (Karimi et al., 2005) to generate the deletion vector following the conditions described by the manufacturer (Invitrogen, Carlsbad, CA). *Agrobacterium tumefaciens*‐mediated transformation was performed based on a previous protocol for *C. rosea* (Utermark & Karlovsky, 2008). Transformed strains were selected on plates containing hygromycin (200 μg/ml). Validation of homologous integration of the deletion cassette in putative transformants was performed using a PCR screening approach as described before (Dubey, et al., 2013; Dubey, et al., 2013; Dubey et al., 2012, 2014a, 2014b). Primers specific to the hygromycin cassette (Dubey, et al., 2013; Dubey, et al., 2013; Dubey et al., 2012, 2014a, 2014b), primers flanking the deletion cassette (abcG6ko F/ abcG6ko R) and combinations of the different primer sets were used (Figure S1). Putative transformants were tested for mitotic stability as described previously (Dubey, et al., 2013; Dubey, et al., 2013; Dubey et al., 2012). Mitotically stable colonies were purified by two rounds of single spore isolation. RT‐PCR analysis was conducted on wild‐type and deletion strains using RevertAid premium reverse transcriptase (Fermentas, St. Leon‐Rot, Germany) and a primer pair specific for *abcG6*.

### 2.11 Phenotypic analyses of gene deletion strains

For growth rate analysis, a 3‐mm‐diameter agar plug with actively growing mycelium was transferred to solid CZ medium or CZ medium supplemented with zearalenone, Apron, Amistar, Chipco green, Cantus or Teldor at the concentrations used for gene expression analysis, except for zearalenone (50 μg/ml) and Teldor (5,000 μg/ml of fenhexamid). Colony diameter was measured in triplicates after three days of growth at 25°C. The concentration of chemicals used for phenotypic analysis in this study was based on our previously published results (Dubey et al., 2014a, 2016). Antagonistic behaviour of *C. rosea* wild‐type and Δ*abcG6* strains against *B. cinerea* and *F. graminearum* was tested in five biological replicates using an in vitro plate confrontation assay and a culture filtrate assay, as described previously (Dubey et al., 2014a, 2016).

In vivo biocontrol of fusarium foot rot disease on wheat and in vitro antagonism of *C*. *rosea* wild‐type and deletion strains were tested as described above. All phenotypic analyses were performed with five independent *abcG6* deletion strains (16A, 16B, 30A, 53A and 53B) in order to confirm that the observed phenotypes were attributed to deletion of *abcG6*, and not to ectopic insertions, if any, of the deletion cassette.

### 2.12 Statistical analysis

Phenotypic data were analysed by analysis of variance (ANOVA) using a general linear model approach implemented in Minitab^®^ Statistical Software version 18.1 (Minitab Inc., State College, PA). Pairwise comparisons were performed using the Tukey‐Kramer or Fisher's least significant difference (LSD) method at the 95% significance level. Gene expression data were analysed by Student's *t* test implemented in Excel (Microsoft).

## 3 RESULTS

### 3.1 Genome sequencing and identification of Clonostachys strains

As part of a large‐scale *C. rosea* genome sequencing initiative (Broberg et al., 2018), genomes of ten strains, previously identified as *C. rosea* based on morphological characters, displayed a low overall alignment rate (<60%) of Illumina HiSeqX reads towards the *C. rosea* strain IK726 reference genome (Table 1). Based on the species identity of these strains (see below), we also selected *C. solani* strain 1703 for genome sequencing; PacBio sequencing generated 54.5 Mbp sequence data across 108 contigs with an N50 of 1.8 Mbp (Table 1). Sequence data for all eleven *Clonostachys* strains were deposited at EMBL European Nucleotide Archive (ENA) under accession PRJEB32493.

**Figure 1:**
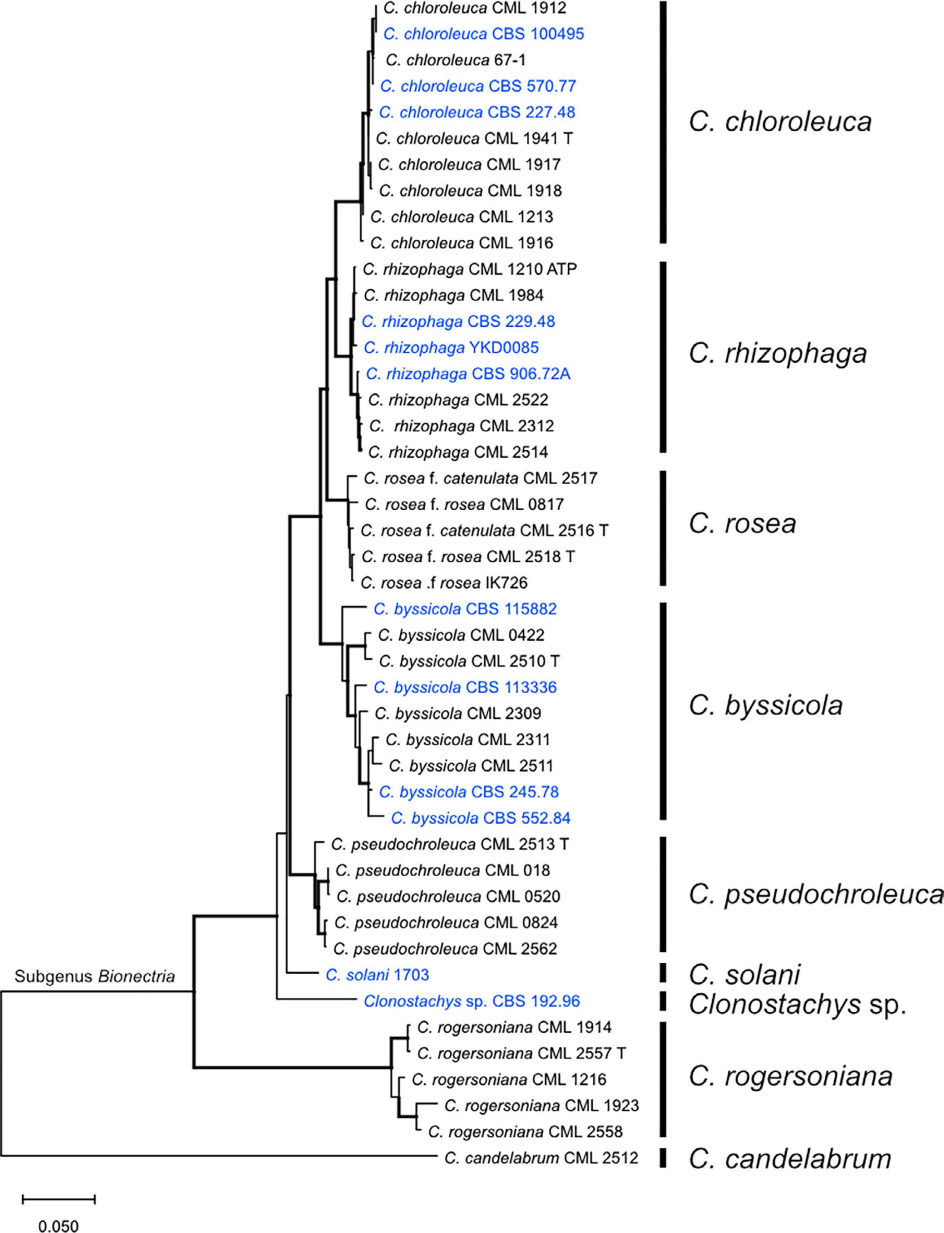
Maximum likelihood tree showing phylogenetic relatedness of selected species of *Clonostachys* subgenus *Bionectria*. The tree is rooted with *C. candelabrum* (*Clonostachys* subgenus *Epiphloea*). Tree is based on concatenated, partial *acl1*, *rpb1*, *tef1* and *tub* gene exon and intron sequences. Bootstrap branch support values (≥70%) based on 1,000 iterations are indicated by bold branches. Sequence identifiers include species and strain ID. The bar marker indicates average number of substitutions per site. Sequence identifiers in blue colour indicate new species name assignments based on the results from the current work. Letter T indicates ex‐type strains

Phylogenetic analyses of concatenated *acl1*, *tef1*, *rpb1* and *tub* partial sequences including gene exons and introns were used to identify here studied strains to species level, together with strains IK726 (Broberg et al., 2018; Karlsson et al., 2015), YKD0085 (Liu et al., 2016), 67‐1 (Sun et al., 2015a) and a *Clonostachys* spp. reference set from Moreira et al. (2016). The analysis of the concatenated dataset identified strains CBS 115882, CBS 113336, CBS 245.78 and CBS 552.84 as *C. byssicola* (99% bootstrap support), CBS 227.48, CBS 570.77, CBS 100495 and 67‐1 as *C. chloroleuca* (99% bootstrap support) and CBS 229.48, CBS 906.72A and YKD0085 as *C. rhizophaga* (99% bootstrap support) (Figure 1). Strain CBS 192.96 from Papua New Guinea was identified as an undescribed *Clonostachys* sp. accommodating a unique lineage within *Clonostachys* subgenus *Bionectria*. The topologies of individual gene trees supported monophyly of the species clades inferred by the concatenated dataset (Supporting Information Figure S2). However, strains identified as *C. rhizophaga* clustered in two polyphyletic subgroups in the *tef1* tree and the *tub* sequence‐based analysis inferred *C. byssicola* as a paraphyletic group with respect to *C. chloroleuca* and *C. rhizophaga* (Figure S2). Macroscopic and microscopic analyses of morphological characters of strains 1703, CBS 245.78, CBS 906.72A, CBS 570.77 and CBS 192.96 did not contradict the phylogenetic species identification.

### 3.2 *Clonostachys* biocontrol of fusarium foot rot and antagonism of *Fusarium graminearum*

**Figure 2:**
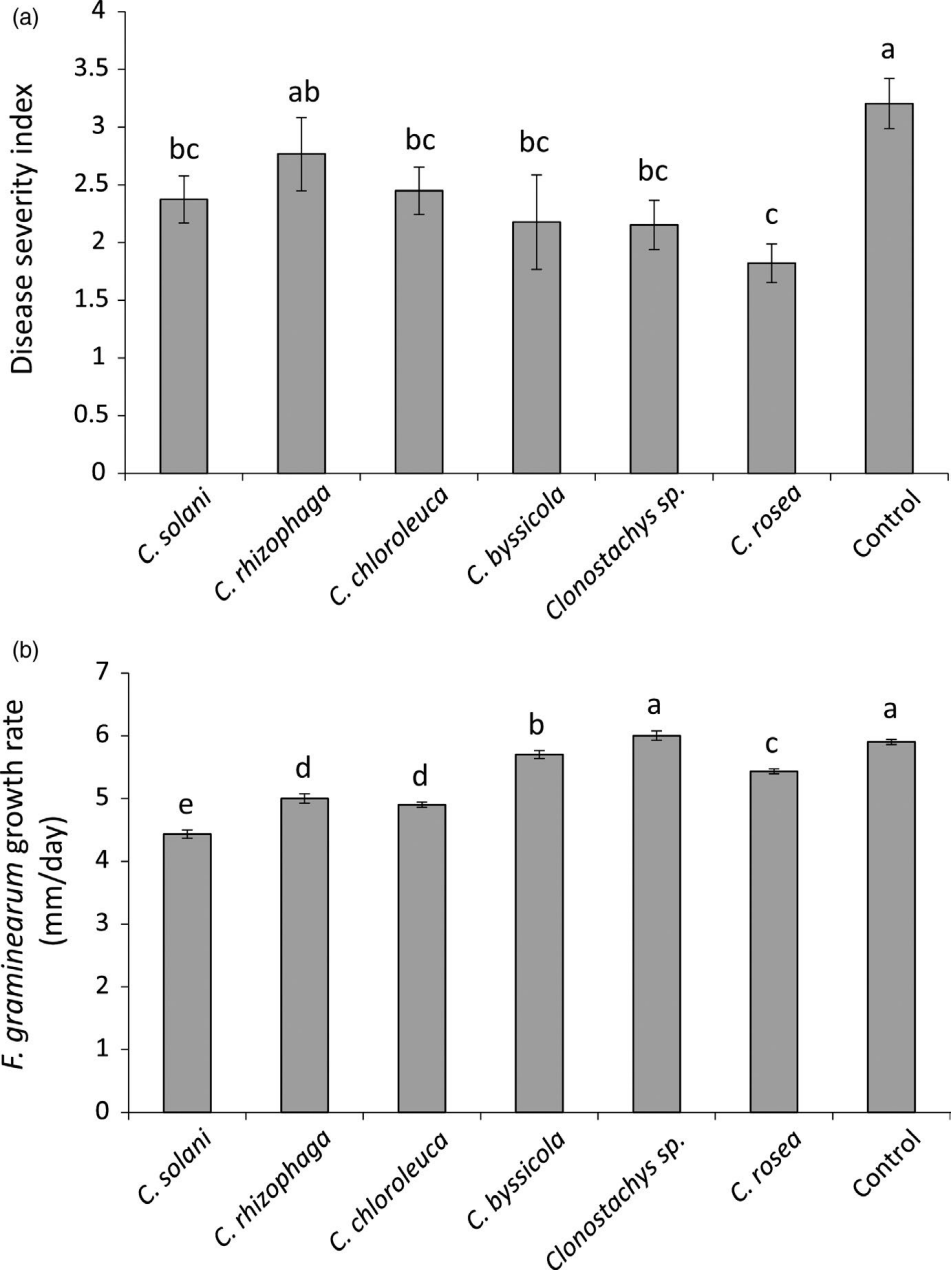
Biocontrol and antagonism of *Clonostachys* species. (A) Wheat seeds were coated with *Clonostachys* spp. conidia and planted in moist sand together with a *F. graminearum* agar plug. Seedlings were harvested three weeks postinoculation and disease symptoms were scored on a 0 to 4 scale. The experiment was performed in five biological replicates with 12–15 plants in each replicate. (B) Mycelial agar plugs of *Clonostachys* spp. and *F. graminearum* were inoculated at opposite sides of a 9‐cm PDA Petri plate. Mycelial growth was measured in five biological replicates. Different letters indicate statistically significant differences (*p* ≤ .05) between treatments based on Fisher's exact test. Error bars represent standard error based on five biological replicates. Strains included were *C. solani* 1703, *C. byssicola* CBS 245.78, *C. rhizophaga* CBS 906.72A, *C. chloroleuca* CBS 570.77, *Clonostachys* sp. CBS 192.96 and *C. rosea* IK726

Strains *C. solani* 1703, *C. byssicola* CBS 245.78, *C. rhizophaga* CBS 906.72A, *C. chloroleuca* CBS 570.77, *Clonostachys* sp. CBS 192.96 and *C. rosea* IK726 were evaluated for their ability to protect wheat seedlings against fusarium foot rot disease caused by *F. graminearum* strain PH‐1 in a climate chamber experiment. All strains, with the exception of *C. rhizophaga*, were able to significantly (*p* ≤ .05) reduce disease symptoms on wheat seedlings by between 24% and 42% (Figure 2a).

Dual culture confrontation assays were used to evaluate in vitro antagonism of *Clonostachys* strains towards *F. graminearum*. All strains, with the exception of *Clonostachys* sp. CBS 192.96, were able to reduce (*p* ≤ .05) growth rate of *F. graminearum* (Figure 2b). Strains differed in their ability to reduce growth rate of *F. graminearum*, with *C. solani* 1703 being the most efficient, followed by *C. rhizophaga* CBS 906.72A, *C. chloroleuca* CBS 570.77, *C. rosea* IK726 and *C. byssicola* CBS 245.78 (Figure 2b).

### 3.3 Comparative genomics of *Clonostachys* species

Newly generated genomes ranged in size from 53.0 Mbp for *Clonostachys* sp. CBS 192.96 to 61.1 Mbp for *C. chloroleuca* CBS 570.77 (Table 1). The genome of *C. rosea* IK726 was reported previously to amount to 70.7 Mbp (Broberg et al., 2018). The here performed comparative genome analysis included newly generated genomes of *C. byssicola* CBS 245.78, *C. chloroleuca* CBS 570.77, *C. rhizophaga* CBS 906.72A, *C. solani* 1703 and *Clonostachys* sp. CBS 192.96, and available reference genomes of *C. chloroleuca* 67‐1, *C. rhizophaga* YKD0085, *C. rosea* IK726, *F. graminearum* PH‐1, *F. verticilloides* 7,600, *Neocosmospora solani* 77‐13‐4, *T. atroviride* IMI 206,040, *T. reesei* QM6a, *T. virens* Gv29‐8 and *Neurospora crassa* OR74A. For comparative purposes, all genomes were annotated using the same in‐house developed gene prediction pipeline. The *Clonostachys* species displayed the highest numbers of predicted genes, ranging from 18,093 (*C. solani*) to 19,658 (*C. chloroleuca*) (Table 1), compared to *Fusarium* (11,994 in *F. graminearum* and 14,286 in *F. verticillioides*), *N. solani* (16,149 genes) and *Trichoderma* (9,566 in *T. reesei*, 11,541 in *T. atroviride* and 12,495 genes in *T. virens*). *Clonostachys chloroleuca* 67‐1 had 18,500 predicted genes, while *C. rhizophaga* YKD0085 had 18,401 and *N. crassa* 9,710 genes. *Clonostachys rosea* IK726 was reported previously to contain 21,246 genes (predicted with the same annotation pipeline) (Broberg et al., 2018).

The proportions of the total DNA bp length of exons, introns, simple repeats and low complexity regions were similar between the annotated *Clonostachys* genomes (Figure S3). However, there was a difference in the genome proportion of dispersed repeats between species. Highest proportion of dispersed repeats were seen in *C. rosea* (13.4%), followed by 6.2%–6.7% in *C. chloroleuca* and *C. rhizophaga* and 0.8%–2.8% in *C. byssicola*, *C. solani* and *Clonostachys* sp (Figure S3).

Different categories of repeat elements were analysed for the presence of signs indicating an active RIP mechanism, a genome‐defence mechanism against transposon replication in certain fungi (Hane et al., 2014). A distorted C:G to T:A DNA base pair ratio defined as a composite RIP index (CRI)> 1, providing strong evidence for an active RIP mechanism, was detected for at least one repeat element category in *C. byssicola*, *C. chloroleuca*, *C. rhizophaga* and *C. solani*, but not in *C. rosea* and *Clonostachys* sp. (Table 3).

**Table 3:** Composite RIP index^a^ of categories of repeat elements in *Clonostachys* species

Repeat category	*C. rhizophaga*	*C. chloroleuca*	*C. rosea*	*C. byssicola*	*C. solani*	*Clonostachys* sp.
Unknown	0.5850	−0.0809	−0.0473	−0.0694	0.1463	−0.4019
LTR/Gypsy	**1.0184**	**1.1248**	0.5869	**1.3281**	**1.1970**	−0.5875
rRNA	0.3663	0.4466	−0.1467	0.4033	−0.0875	0.4464
LTR/Copia	**1.3243**	**1.0776**	0.3140	−0.9197	−0.6369	−0.8137
LTR	**1.2374**	n.a.	0.0740	n.a.	n.a.	−1.0000
DNA/TcMar‐Fot1	0.8819	0.7562	−0.2726	−0.8366	**1.1644**	−0.4781
RC/Helitron	−0.4219	−1.4167	−0.5168	−1.5545	−1.7500	0.0000
DNA/hAT‐Ac	−0.9250	−0.7593	−0.7391	n.a.	−0.8329	−0.5000
DNA/hAT‐Restless	−0.4604	−0.4652	−0.6221	n.a.	n.a.	0.6095
DNA/PiggyBac	−0.1134	−0.0658	−0.2214	0.6167	−0.8077	−0.1875

Abbreviation: n.a., not applicable.

^a^ A composite RIP index above 1 (marked in bold) provides strong evidence for an active RIP mechanism.

### 3.4 Analysis of evolution of gene family composition

OrthoFinder was used to group predicted proteins from all species into orthogroups. A total of 19,191 orthogroups were predicted, of which 5,720 contained members from all included species. The whole‐proteome phylogenetic consensus analysis of all orthogroups confirmed the relative relatedness of targeted species bases on phylogenetic marker genes in Figure 1, the identification of strain YKD0085 as *C. rhizophaga* and strain 67‐1 as *C. chloroleuca* (Table S4). This species tree was used in a comparative gene family evolution analysis using the CAFE software, together with predicted orthogroups and additional gene families predicted to encode transport proteins (based on TCDB), carbohydrate‐active enzymes (dbCAN) and secondary metabolite biosynthesis proteins (antiSMASH).

A total of 632 out of 5,791 orthogroups were shown to evolve nonrandomly (*p* ≤ .05) between species (Table S4). Thirty‐three orthogroups were identified as evolving nonrandomly in the lineage leading to *Clonostachys* subgenus *Bionectria* (Table 4), while 265, 55, 44, 43, 26 and 20 orthogroups were evolving nonrandomly in *C. rosea*, *C. byssicola*, *C. solani*, *C. chloroleuca*, *Clonostachys* sp. and *C. rhizophaga*, respectively. Kinfin annotation showed that orthogroups predicted to encode, among others, PKS, nonribosomal peptide synthases (NRPS), CYP, ABC transporters, MFS transporters, oxidoreductases and ankyrin‐repeat proteins that were expanded in the ancestor to *Clonostachys* subgenus *Bionectria* (Table 4).

**Table 4:** Gene families^a^ expanded in the ancestor to the subgenus *Bionectria*

Gene family ID	*p*‐value	Change^b^	Annotation^c^
OG0000014	.000	13	‐
OG0000020	.000	11	‐
OG0000028	.000	10	Protein of unknown function DUF3433
OG0000001	.001	15	CHAT domain protein
OG0000017	.002	10	Protein kinase domain protein
OG0000050	.002	7	Protein of unknown function DUF3632
OG0000016	.004	9	Polyketide synthase
OG0000066	.005	6	‐
OG0000005	.009	10	ABC transporter
OG0000272	.010	5	‐
OG0000293	.010	5	Amino acid permease
OG0000049	.012	6	Nonribosomal peptide synthetase
OG0000047	.012	6	Amino acid transporter
OG0000359	.020	4	‐
OG0000120	.020	4	‐
OG0000202	.020	4	MFS transporter
OG0000176	.020	4	Ankyrin repeat‐containing protein
OG0000006	.022	8	Cytochrome P450 monooxygenase
OG0000094	.023	5	FMN‐dependent dehydrogenase
OG0000585	.042	3	‐
OG0000391	.042	3	MFS transporter
OG0000809	.042	3	‐
OG0000305	.042	3	‐
OG0000626	.042	3	Cupin 1 protein
OG0000329	.042	3	FAD‐dependent oxidoreductase
OG0000189	.042	3	ATPase, AAA‐type protein
OG0000674	.042	3	‐
OG0000356	.042	3	‐
OG0000238	.042	3	Protein of unknown function DUF3425
OG0000179	.042	3	‐
OG0000102	.043	4	FAD‐dependent oxidoreductase
OG0000071	.043	4	Amino acid permease
OG0000004	.049	8	Aldehyde dehydrogenase

^a^ Gene families predicted with OrthoFinder ver. 1.1.8.

^b^ Gene family size change as compared with the most recent ancestor.

^c^ Annotation based on Kinfin using protein domain sequence coverage towards InterPro categories.

The OG0000016 PKS gene family was significantly (*p* = .004) expanded in the ancestor of *Clonostachys* subgenus *Bionectria* (12 genes) and significantly (*p* < .001) contracted in *C. byssicola* with six genes. A closer investigation confirmed that OG0000016 included not only the previously reported *C. rosea* lovastatin diketide‐type reducing polyketides *pks5*, *pks6*, *pks7*, *pks9*, *pks12*, *pks13*, *pks21*, *pks25* and *pks26* but also three other genes (protein IDs CRV2T00014019, CRV2T00014034, CRV2T00014022, Table S5) not previously reported in *C. rosea* ver. 1 genome (Karlsson et al., 2015). A CAFE analysis of secondary metabolite biosynthesis gene families was done using antiSMASH, which identified type I PKS as expanded (*p* = .024) in the ancestor of *Clonostachys* subgenus *Bionectria* (Table S4). As type I PKS includes reducing PKS, the two analyses were congruent. A small, but significant (*p* = .020), loss of two type I PKS genes (from 13 to 11) was also identified in *C. solani*.

Further evidence for the importance of secondary metabolite biosynthesis for *Clonostachys* spp. comes from the expansion (*p* = .012) of the OG0000049 gene family, predicted to encode NRPS, in the ancestor of *Clonostachys* subgenus *Bionectria* (Table 4). A significant (*p* = .023) loss of two genes in OG0000049 was also identified in *C. chloroleuca* (Table S4). OG0000049 included the previously reported *C. rosea* putative peptaibol synthetase NRPS genes *nps4*, *nps5*, *nps7*, *nps9*, *nps12*, *nps15* and *nps16* (Karlsson et al., 2015) and two not previously reported genes (CRV2T00021335 and CRV2T00011221, Table S5). Analysis of antiSMASH gene families identified a small NRPS gene loss (from 13 to 11, *p* = .045) in *C. rhizophaga* (Table S4).

Four CYP gene families were evolving nonrandomly (*p* ≤ .05): OG0000006 expanded in the ancestor of *Clonostachys* subgenus *Bionectria* and contracted in *C. solani*, OG0001128 and OG0014233 expanded in *C. rosea*, and OG0000345 contracted in *C. solani* (Table S4). Members in OG0000006 displayed sequence similarity to genes encoding proteins involved in secondary metabolite biosynthesis (Table S5) and included *cyp1* (CRV2T00003572) in *C. rosea* with similarity to an isotrichodermin C‐15 hydroxylase (Nygren et al., 2018). Members in OG0001128, OG0014233 and OG0000345 also displayed sequence similarity towards secondary metabolite biosynthesis genes (Table S5).

### 3.5 Evolution of carbohydrate‐active enzyme gene family composition

CAFE analysis of carbohydrate‐active enzyme gene family composition predicted by dbCAN identified 14 families evolving nonrandomly in *Clonostachys* species (Table 5). Most *Clonostachys* species contained high numbers of AA9 lytic polysaccharide monooxygenases. A significant (*p* = .003) increase (from 12 to 27 genes) in the ancestor of *Clonostachys* subgenus *Bionectria* was followed by further gene gains in *C. chloroleuca* (*p* = .018) and gene losses in *C. solani* (*p* < .001) (Table 5). All *Clonostachys* species also contained high numbers of AA3 glucose‐methanol‐choline oxidoreductases (17‐19 genes) compared with *Fusarium* (7‐8 genes) and *Trichoderma* (4‐7 genes). The AA7 oligosaccharide oxidase gene family was expanded in *C. rosea* (*p* < .001) but contracted in *C. chloroleuca* and *C. rhizophaga* (*p* ≤ .027) (Table 5). Another hallmark seen in all tested *Clonostachys* species was expansions of gene families with putative activity (or binding) towards xylan (carbohydrate esterase [CE] family 3, glycoside hydrolase [GH] family 3, GH16 and GH43) and rhamnose/pectin (carbohydrate‐binding module [CBM] family 67 and GH78) substrates (Table 5).

**Table 5:** Gene numbers in nonrandomly^a^ evolving *Clonostachys* carbohydrate‐active enzyme gene families

Gene family^b^	Description	C. rhi	C. chl	C. ros	C. bys	C. sol	Clono sp.	Bionectria
AA3	Glucose–methanol–choline oxidoreductase	18	19	19	18	18	17	17
AA3_3	Alcohol oxidase	6	5	5	4	3	5	5
AA7	Gluco‐ and chitooligosaccharide oxidase	28	26	44	34	31	36	32
AA9	Lytic polysaccharide monooxygenase	31	35	29	33	18	29	27
CBM67	L‐rhamnose binding module	7	9	7	6	7	13	8
CE1	Esterase (variable substrates)	10	11	12	9	9	10	10
CE3	Acetyl xylan esterase	13	13	12	11	16	15	14
CE10	Esterase (noncarbohydrate substrates)	72	72	77	74	73	79	73
GH3	Glucanase, xylanase	32	33	37	34	30	30	31
GH16	Glucanase, xylanase	26	28	31	26	25	26	26
GH43_26	Xylanase, α‐L‐arabinofuranosidase	11	11	11	11	12	12	11
GH43_36	Xylanase	4	6	3	4	3	3	3
GH78	α‐L‐rhamnosidase	12	15	10	11	12	17	13
GT1	Glycosyltransferase 1	16	15	19	15	18	14	15

Species abbreviations: C. rhi = *Clonostachys rhizophaga*, C. chl = *Clonostachys chloroleuca*, C. ros = *Clonostachys rosea*, C. bys = *Clonostachys byssicola*, C. sol = *Clonostachys solani*, Clono sp. = *Clonostachys* sp. (unknown species), Bionectria = The ancestor species to the subgenus *Bionectria*.

^a^ Gene numbers boxed in black indicates a significant (*p* ≤ .05) expansion, while gene numbers boxed in grey indicates a significant (*p* ≤ .05) contraction of gene family size compared with the most recent ancestor.

^b^ Carbohydrate‐active enzyme gene family classification is based on the Carbohydrate‐Active enZYmes Database (CAZy).

### 3.6 Evolution of membrane transporter gene family composition

CAFE analysis of membrane transporter gene family composition predicted by TCDB identified 20 nonrandomly (*p* ≤ .05) evolving families in the tested *Clonostachys* species (Table 6). The highest number of changes was detected in *C. rosea* with 11 expansions and two contractions, followed by *C. byssicola* with seven contractions and one expansion (Table 6). Six families evolving under selection for increased gene copy number were identified in the ancestor of *Clonostachys* subgenus *Bionectria* and annotation suggested their involvement in various house‐keeping functions, including transport of fatty acids (4.C.1), ions (8.A.5), amino acids (9.A.70), small organic compounds (2.A.1.13) and recycling of membrane proteins (9.A.3). The only exception was the drug:H^+^ antiporter‐2 MFS transporter family (2.A.1.3) involved in drug resistance. The expansions of 9.A.3, 9.A.70 and 2.A.1.3 in the subgenus *Bionectria* ancestor were followed by additional gene gains in *C. rosea* for 9.A.3 and 2.A.1.3, and for *C. rosea* and *C. byssicola* for 9.A.70 (Table 6). Significant contractions of the 2.A.1.3 drug resistance MFS transporter family were identified in *T. reesei* (*p* = .004, from 37 to 22 genes) and *N. crassa* (*p* = .006, from 36 to 18 genes) (Table S4).

**Table 6:** Gene numbers in nonrandomly^a^ evolving *Clonostachys* transporter gene families

Gene family^b^	Description	C. rhi	C. chl	C. ros	C. bys	C. sol	Clono sp.	Bionectria
1.B.12	The Autotransporter‐1 Family	3	2	6	3	4	3	3
1.C.104	The Heterokaryon Incompatibility Prion/Amyloid Protein Family	4	4	5	1	2	3	3
1.C.105	The Bacillus thuringiensis Vegetative Insecticidal Protein‐3 (Vip‐3) Family	3	5	5	6	6	5	5
1.C.113	The Hly III Family	5	5	8	4	6	4	5
1.C.63	The α‐Latrotoxin Family	3	4	2	3	7	8	5
3.A.23	The Type VI Symbiosis/Virulence Secretory Pathway Family	8	8	11	5	7	2	5
3.A.9	The Chloroplast Envelope Protein Translocase Family	24	29	37	30	27	27	27
4.C.1	The Fatty Acid Transporter Family	64	67	68	71	68	77	69
8.A.23	The Basigin Family	3	2	5	1	5	2	3
8.A.32	The β‐Amyloid Cleaving Enzyme Family	5	6	3	6	5	6	5
8.A.5	The Voltage‐gated K + Channel β‐subunit Family	60	61	60	65	57	65	60
8.A.51	The Dipeptidyl‐aminopeptidase‐like Protein 6 beta subunit of Kv4 channels Family	15	15	23	9	11	11	11
8.A.95	The Transmembrane and TPR Repeat‐containing Protein 3 Family	6	6	11	2	4	1	3
9.A.3	The Sorting Nexin 27 Retromer Assembly Apparatus for Recycling Integral Membrane Proteins Family	75	75	108	81	67	57	65
9.A.62	The AAA‐ATPase, Bcs1 Family	10	11	13	6	9	8	9
9.A.70	The Aspartate Amino Transferase Family	10	12	17	15	10	11	11
9.B.106	The Pock Size‐determining Protein Family	64	64	67	56	65	61	61
MFS transporters								
2.A.1.13	The Monocarboxylate Transporter Family	59	55	55	60	53	57	54
2.A.1.3	The Drug:H + Antiporter‐2 Family	89	84	106	87	79	80	81
2.A.1.9	The Phosphate: H + Symporter Family	10	12	6	7	7	7	8
ABC transporters								
ABC‐B4	Multidrug resistance transporters	4	3	11	3	7	5	5
ABC‐C5	Multidrug resistance‐associated transporters	14	14	17	13	14	13	13
ABC‐G1	Pleiotropic drug resistance transporters	20	19	16	19	15	18	17

Species abbreviations: C. rhi = *Clonostachys rhizophaga*, C. chl = *Clonostachys chloroleuca*, C. ros = *Clonostachys rosea*, C. bys = *Clonostachys byssicola*, C. sol = *Clonostachys solani*, Clono sp. = *Clonostachys* sp. (unknown species), Bionectria = The ancestor species to the subgenus *Bionectria*.

^a^ Gene numbers boxed in black indicates a significant (*p* ≤ .05) expansion, while gene numbers boxed in grey indicates a significant (*p* ≤ .05) contraction of gene family size compared with the most recent ancestor.

^b^ Transporter classification is based on the Transporter Classification Database (TCDB) or based on Kovalchuk and Driessen (2010) in the case of ABC transporters.

Members of five transporter gene families were classified as virulence factors in biotic interactions: the 1.B.12 autotransporter‐1 family, the pore‐forming 1.C.105 Vip‐3, 1.C.113 Hly III and 1.C.63 latrotoxin families, and the 3.A.23 type IV symbiosis/virulence secretory pathway family. Three of these, 1.B.12, 1.C.105 and 1.C.113, were specifically expanded (*p* ≤ .009) in *C. rosea* (Table 6). The 1.C.63 latrotoxin gene family, predicted to encode cytotoxic pore‐forming proteins, was expanded (*p* = .031) in *T. virens* (from two to five genes), and in *Clonostachys* sp. CBS 192.96 (*p* = .050), from five to eight genes (Table 6, Supporting Information Table S4). The second most basal *Clonostachys* species in the current comparison, *C. solani*, also had a high number (seven) of 1.C.63 genes, but this was followed by a significant (*p* = .009) loss in the lineage leading to the other studied species of subgenus *Bionectria* (Table S4).

Eight ABC transporter gene families, ABC‐B3 (full‐sized), ABC‐B3 (half‐sized), ABC‐B4, ABC‐C5, ABC‐G1, ABC‐G3, ABC‐G5 and ABC‐G6, evolved nonrandomly (*p* ≤ .040, Supporting Information Table S4). Two families involved changes in *C. rosea*: a seven gene increase (*p* < .001) in ABC‐B4 multidrug resistance transporters and a three gene increase (*p* = .018) in ABC‐C5 multidrug resistance‐related transporters (Table 6). The pleiotropic drug resistance transporter family ABC‐G1 displayed a continuous increase in the lineages leading to *Clonostachys* subgenus *Bionectria*. The increase consisted of a five gene increase (*p* < .001) in the lineage leading to the *Bionectriaceae* and *Nectriaceae*, followed by a nine gene increase (*p* = .015) in the subgenus *Bionectria* lineage, resulting in high ABC‐G1 gene numbers in all analysed *Clonostachys* species (Table 6, Table S4).

**Figure 3:**
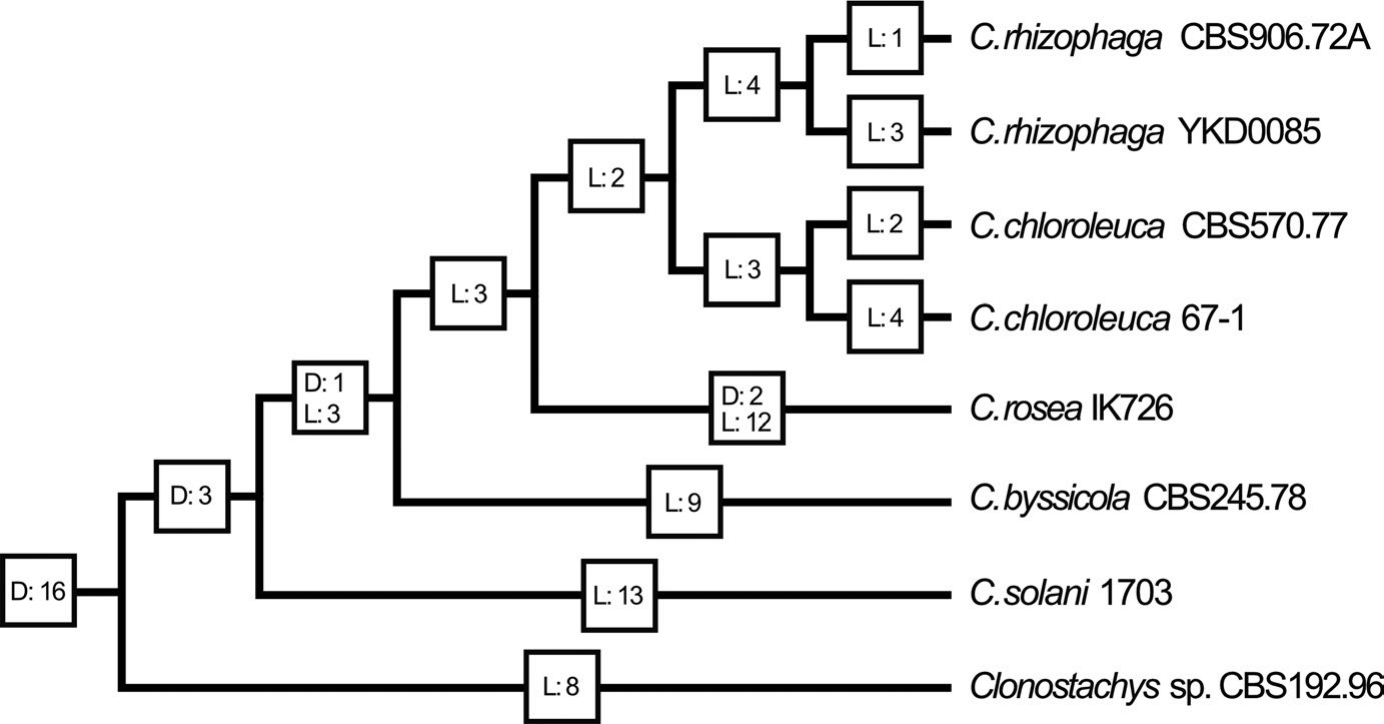
Reconciliation of species and pleiotropic drug resistance transporter gene trees. A summary of the reconciliation analysis of the *Clonostachys* spp. species tree and the ABC‐G1 pleiotropic drug resistance transporter gene tree using NOTUNG (Figure S5) is presented. D = number of gene duplications associated with a branch, L = number of gene losses associated with a branch. Species and strain ID are given

Phylogenetic analysis of the *Clonostachys* ABC‐G1 gene family revealed low resolution among the deeper branches, and incongruence with the species phylogeny in several cases (Figure S4). Reconciliation analysis of the *Clonostachys* species tree and the ABC‐G1 gene tree using Notung showed high levels of lineage‐specific gene losses, but also a few gene duplications (Figure 3, Figure S5). The number of ABC‐G1 gene losses in extant *Clonostachys* species varied from three in *C. chloroleuca* to thirteen in *C. solani*, while two gene duplications were detected in *C. rosea* (Figure 3).

### 3.7 Structural divergence of ABC‐G1 pleiotropic drug resistance transporters

**Figure 4:**
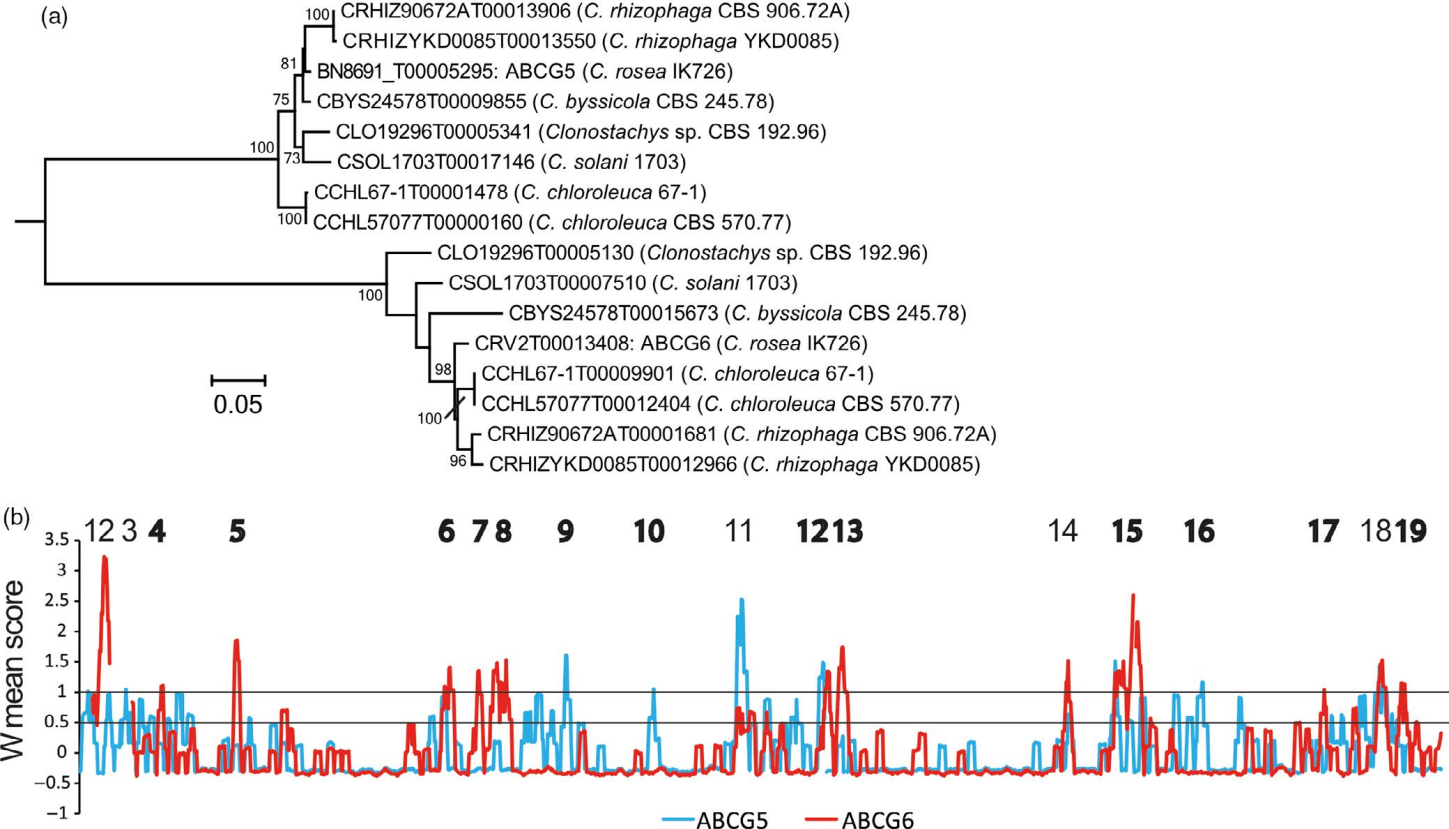
Sequence analysis of pleiotropic drug resistance transporters. (a) Phylogenetic relationships of two paralogous ABC‐G1 pleiotropic drug resistance transporter groups in *Clonostachys* (from Figure S4). Predicted ABC transporter protein sequences were aligned with MUSCLE and phylogenetic analysis was performed using maximum likelihood methods implemented in MEGA Statistical support for branches was assessed by 500 bootstrap resampling. (b) Reverse conservation analysis of ABC‐G1 groups. Amino acid conservation was estimated using Rate4Site, based on a MUSCLE alignment, and plotted as W mean scores in arbitrary units. The blue and red lines represent the ABCG5 and ABCG6 groups, respectively. Regions with signs of functional divergence (W ≥ 1 in one group and W < 1 the other group) are indicated

Having established an ongoing birth‐and‐death evolutionary process of *Clonostachys* ABC‐G1 genes, sequence divergence of paralogs was investigated using RCA. The previously characterized *C. rosea* ABC‐G1 transporter ABCG5 (Dubey et al., 2014a) was reported to be paralogous to *C. rosea* ABCG6 (Karlsson et al., 2015), which was supported by our phylogenetic ABC‐G1 sequence analysis (Figure S4). ABCG5 and ABCG6 were members of two well‐supported phylogenetic groups (bootstrap support = 100%), each containing eight predicted *Clonostachys* proteins (Figure 4a). The RCA analysis identified nineteen regions, labelled 1 through 19, which displayed high amino acid variation (defined by W ≥ 1) (Figure 4b, Table S6). From these, thirteen regions (4, 5, 6, 7, 8, 9, 10, 12, 13, 15, 16, 17 and 19) showed signs of functional divergence between the two groups, defined as high amino acid variation (W ≥ 1) in one group in combination with low variation (W < 0.5) in the other group (Figure 4b, Table S6).

**Figure 5:**
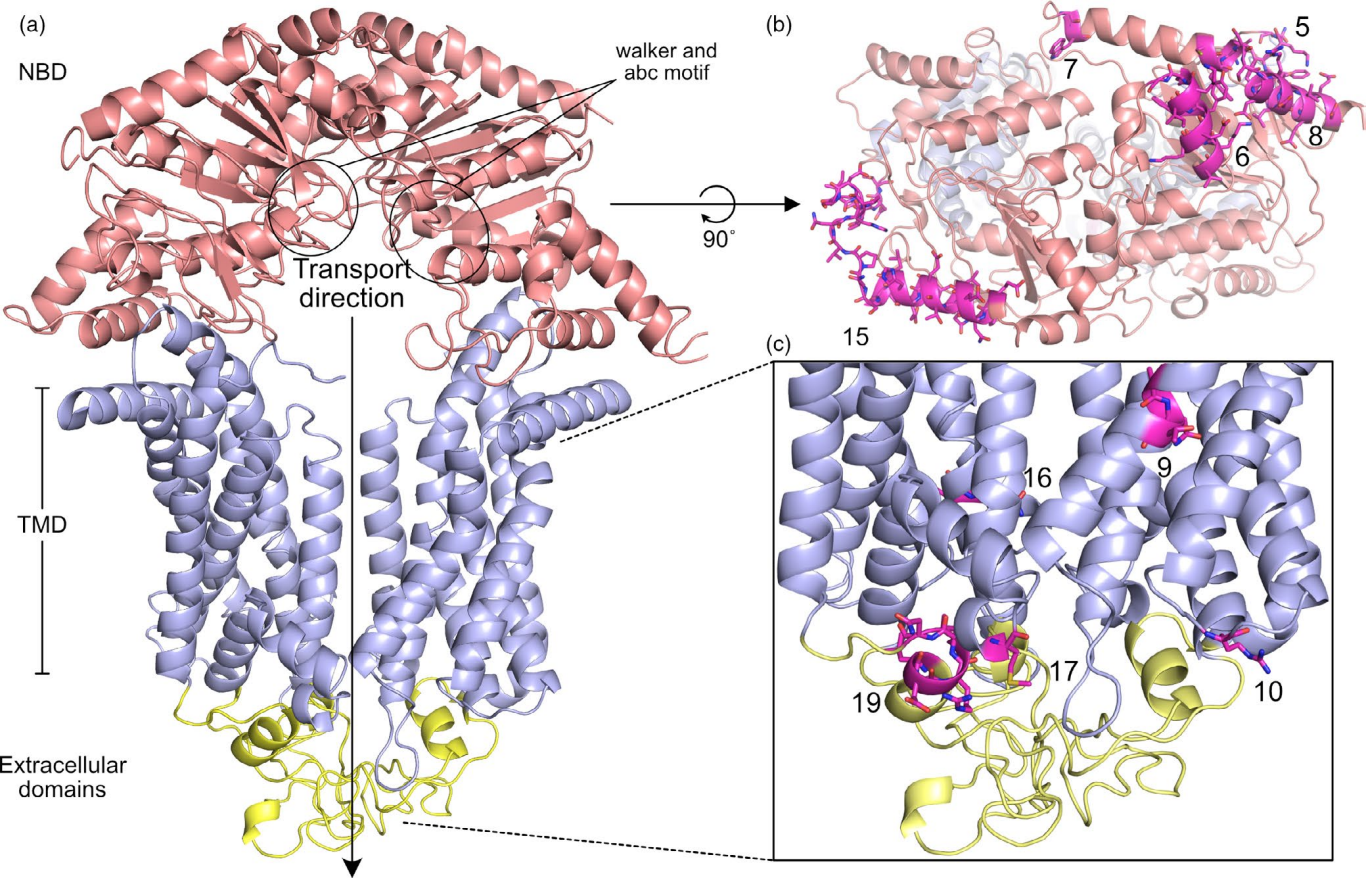
Overview of the predicted ABCG6 structure. (a) The homology model of ABCG6 was generated via the I‐TASSER server. Cytosolic nucleotide‐binding domains (NBDs) are coloured in red, transmembrane domains (TMDs) in blue and extracellular domains in yellow. In the zoomed in sub figures (b) and (c), RCA regions with sequence divergence between the ABCG5 and ABCG6 groups are highlighted in magenta sticks with their corresponding RCA numbers. This highlights the structural clustering of diverging regions near the substrate export tunnel and extracellular domain (c) and in several surface helices at the NBDs (b)

Analyses of conserved protein modules and homology modelling predicted that regions 4, 5, 6, 7 and 8 were located in the long N‐terminal intracellular part containing the first NBD (Figures 4b, 5a and 5b). More specifically, region 4 was located in a predicted ABC_trans_N domain (InterPro ID: IPR029481), while region 6 was bordering a predicted ATPase module (InterPro ID: IPR003593). Regions 12, 13 and 15 were located in the second, C‐terminal NBD, where region 12 bordered a predicted PDR_CDR domain (InterPro ID: IPR010929) (Table S6). Three regions (9, 16 and 17) were located in transmembrane helices 1, 8 and 11, respectively (Figures 4b and 5c, Table S6). Region 10 was located in extracellular loop number 2, while region 19 was located in extracellular loop 6. In addition, regions 11 and 18 with high amino acid variation in both groups were located in extracellular loops 3 and 6, respectively (Table S6). A closer inspection of the sequence alignments of extracellular loops 3 and 6 identified thirteen and fourteen amino acid differences, respectively, which were fixed between the two groups (Figure S6).

The N‐ and C‐terminal ATPase modules of ABCG5 and ABCG6 contained all conserved motifs necessary for a functional ABC transporter, including N‐terminal Walker A1 (GPPGAGCTT), Q‐loop (Q), C‐loop (VSGGE), Walker B1 (LQCWD), D‐loop (N) and H‐loop (Y), and C‐terminal Walker A2 (GVSGAGKT), Q‐loop (Q), C‐loop (LNVEQ), Walker B2 (LLFVD), D‐loop (E) and H‐loop (H) (Table S6).

### 3.8 Gene expression analysis of *abcG6* in *C. rosea*

**Figure 6:**
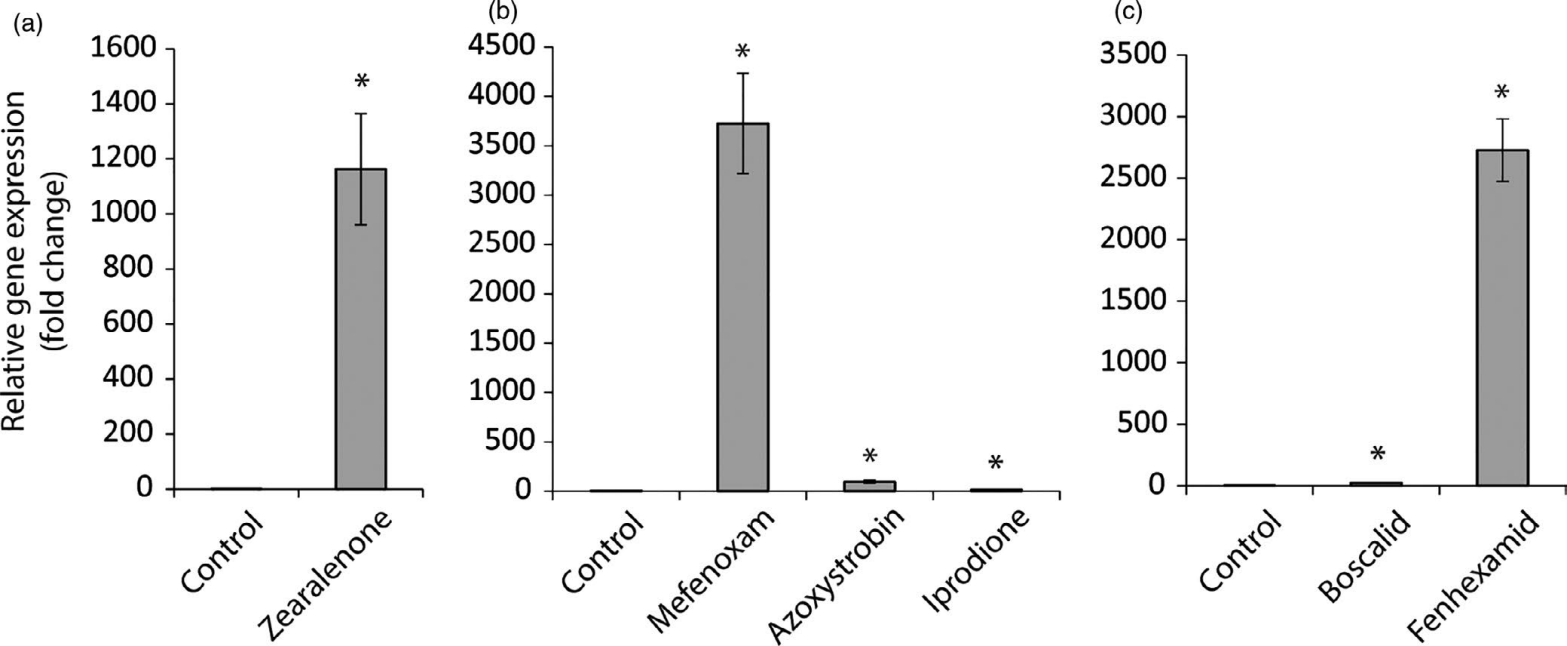
Gene expression analysis of *abcG6*. Expression of *abcG6* in *C. rosea* was estimated with RT‐qPCR during exposure to (a) zearalenone, (b) mefenoxam, azoxystrobin and iprodione, and (c) boscalid and fenhexamid. Relative gene expression was calculated using the 2^‐∆∆Ct^ method, and normalized by β‐tubulin (*tub*) expression. Error bars represent standard error based on five biological replicates. An asterisk indicates a statistically significant difference (*p* ≤ .05) compared with the respective control treatment based on Student's *t* test

Transcript abundance of *abcG6* in *C. rosea* was estimated using RT‐qPCR. Expression of *abcG6* was neither induced during dual plate interaction with *B. cinerea* (*p* = .726) nor *F. graminearum* (*p* = .772), compared with a self‐interaction control. However, exposure of *C. rosea* to the antifungal *Fusarium* mycotoxin zearalenone resulted in an 1121‐fold induction (*p* < .001) of *abcG6* (Figure 6a). Expression of *abcG6* was also induced (*p* ≤ .009) by exposure of *C. rosea* to a range of different fungicides, including azoxystrobin (91‐fold), mefenoxam (3591‐fold), iprodione (13‐fold) (Figure 6b), boscalid (20‐fold) and fenhexamid (2261‐fold) (Figure 6c).

### 3.9 Phenotypic analysis of *abcG6* gene deletion strains

*Agrobacterium tumefaciens*‐mediated transformation was used to replace *abcG6* in *C. rosea* with the hygromycin selection cassette (hygB). Successful gene replacements were confirmed in five mitotically stable mutants by PCR using primers located within the hygB cassette in combination with primers located in regions flanking the construct (Figure S1). RT‐PCR experiments using primers specific to *abcG6* demonstrated complete loss of transcripts in each mutant (Figure S1).

The ability to protect wheat seedlings against fusarium root rot was not compromised in the *abcG6* gene deletion strains, compared with the wild‐type strain (*p* = .225). Dual plate interactions showed no (*p* ≥ .067) differences in growth rate of *B. cinerea* or *F. graminearum* during confrontation with Δ*abcG6* strains in comparison to the wild type. Even after three‐week‐long dual culture interactions, no differences in overgrowth of *B. cinerea* or *F. graminearum* were observed between the wild‐type and the Δ*abcG6* strains. Furthermore, no significant differences (*p* ≥ .307) in biomass production were found between the wild‐type and Δ*abcG6* strains when grown in culture filtrates from *B. cinerea* or *F. graminearum*.

**Figure 7:**
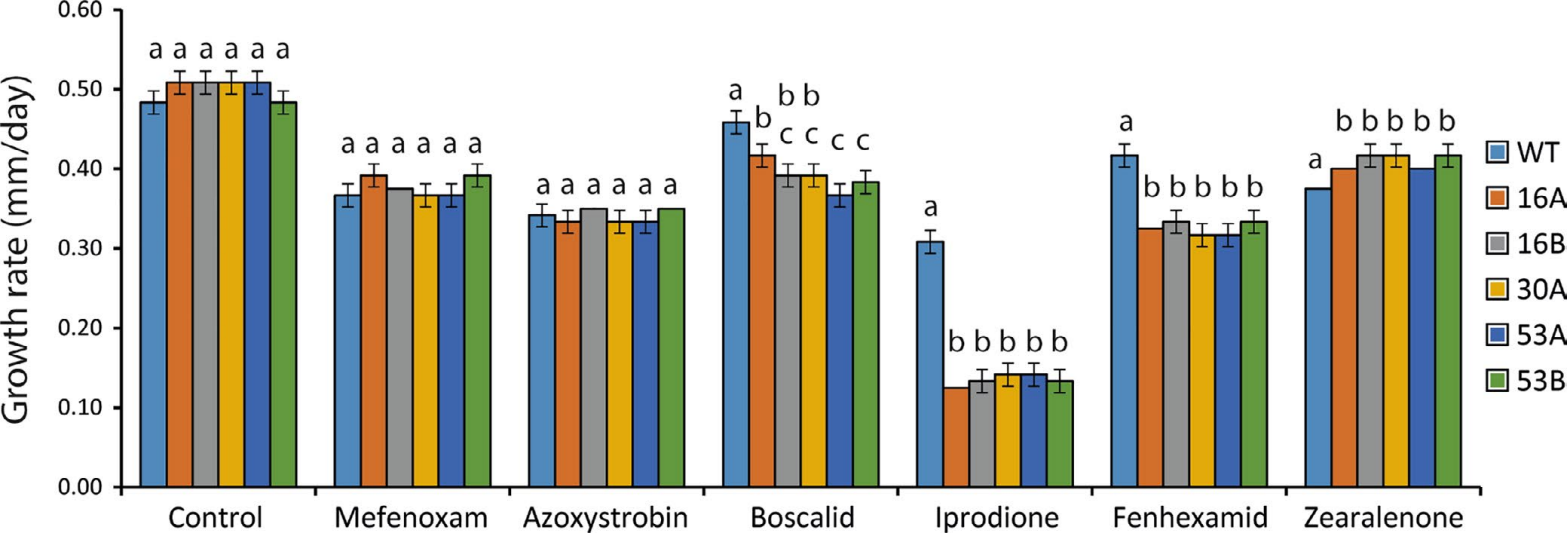
Phenotypic analyses of ∆*abcG6* strains. *Clonostachys rosea* wild‐type (WT) and *abcG6* gene deletion strains (16A, 16B, 30A, 53A and 53B) were inoculated on solid CZ medium (Control) or CZ medium supplemented with fungicides mefenoxam, azoxystrobin, boscalid, iprodione or fenhexamide, or the mycotoxin zearalenone. Mycelial growth was measured in three biological replicates. Error bars represent standard deviation based on three biological replicates. Different letters indicate statistically significant differences (*p* ≤ .05) between treatments based on the Tukey–Kramer test

There were no differences in morphology (Figure S7) or growth rate on CZ medium (*p* = .099, Figure 7) between ∆*abcG6* and wild‐type strains. However, there was a small, 1.1‐fold increase (*p* = .002) in growth rate of the ∆*abcG6* strains when grown on zearalenone‐containing medium, compared with the wild‐type strain (Figure 7). Growth rates of ∆*abcG6* strains were reduced (*p* < .001) on media containing boscalid by 15%, fenhexamid by 22% and iprodione by 56%, while no reduction (*p* ≥ .081) was observed on media containing azoxystrobin or mefenoxam (Figure 7).

## 4 DISCUSSION

In the current work, we set out to investigate factors for distinguishing *C. rosea* as a more efficient BCA from other *Clonostachys* species. We hypothesized that factors contributing to efficient mycoparasitism and polyphagy should enable recognition and exploitation of *C. rosea* as a beneficial agent for controlling plant diseases in crop production. Identification of several presumed *C. rosea* strains as other *Clonostachys* species, together with *C. solani*, provided us with a suitable dataset to perform a comparative genomic investigation of *Clonostachys* subgenus *Bionectria*. Reclassification of fungal strains is common and reflects the continuous development and increasing resolution in fungal taxonomy. One example is *C. rhizophaga* strain YKD0085 that was earlier reported as *C. rosea* (Liu et al., 2016). Already earlier, strain 67‐1 was correctly identified as *C. chloroleuca* (Moreira et al., 2016), although it was originally reported as *C. rosea* (Sun et al., 2015a).

We also show that *C. rosea* is not the only species that can be exploited for biological control. Besides *C. rosea*, *C. solani* 1703, *C. byssicola* CBS 245.78, *C. chloroleuca* CBS 570.77 and *Clonostachys* sp. CBS 192.96 were all able to control fusarium foot rot disease on wheat. This opens up new possibilities with regard to screening programmes to find efficient *Clonostachys* BCAs for incorporation into plant protection strategies in agricultural production, in addition to the few previously reported examples involving *C. chloroleuca* (Sun et al., 2017) and *C. byssicola* (Garcia et al., 2003; Krauss et al., 2006). The fact that most of the tested strains also inhibited growth of *F. graminearum* in dual‐plate assays indicates that antibiosis is one mechanism leading to antagonism and mycoparasitism, consequently contributing to the biocontrol trait. However, it is also clear from our data that there is no linear relationship between in vitro antagonism and biocontrol; for example, *Clonostachys* sp. CBS 192.96 was not able to suppress growth of *F. graminearum* in vitro but could be exploited to efficiently control the disease. This discrepancy between in vitro antagonism and biocontrol is well known within the biocontrol research area (Jensen et al., 2016; Knudsen et al., 1997) and exemplifies the complexity of the biocontrol trait, involving multiple mechanisms depending on the BCA, pathogen, host plant and environment (Harman et al., 2004; Jensen et al., 2017).

We identified several features that relate to evolution of gene family composition that are unique for species of the subgenus *Bionectria*. This lineage exhibits several significant gene family expansions that provide important clues for understanding evolutionary trajectories in *Clonostachys*. Most importantly, expansions of gene families encoding proteins involved in biosynthesis of (PKS, NRPS, CYP) and protection against (ABC and MFS transporters, CYP) secondary metabolites suggest that balancing the levels of secondary metabolites was an important adaptation in the ecological niche (Wapinski et al., 2007) of the subgenus *Bionectria* ancestor. As the nutritional mode of the ancestor of the Hypocreales was hypothesized to be plant‐based (Sung et al., 2008; Zhang et al., 2018), it is tempting to speculate that these drastic evolutionary changes in *Clonostachys* provides an advantage for becoming a generalist, rather than a phytophage specialist. Tolerance to various secondary metabolites from other fungi may in fact be a prerequisite for evolving a mycoparasitic lifestyle.

The effector concept in parasite–host interactions (Kemen, Agler, & Kemen, 2015; Win et al., 2012) predicts that the co‐evolutionary arms race between parasite and host results in effector genes and gene families that evolves under diversifying selection. A broad definition of ecological effectors includes secondary metabolites and cell wall‐degrading enzymes with an effect in intermicrobial interactions (Hogenhout et al., 2009; Kemen et al., 2015). Transcriptomic data from *C. rosea* (Demissie et al., 2018, 2020; Lysøe et al., 2017; Nygren et al., 2018) and *C. chloroleuca* (Sun et al., 2015b, 2020) reported induced expression of PKS, NRPS, CYP, ABC and MFS transporter genes during interactions with fungal prey. Phenotypic analyses of gene deletion strains also demonstrated involvement of PKS (Fatema et al., 2018), NRPS (Iqbal et al., 2019), ABC (Dubey et al., 2014a, 2016) and MFS transporters (Nygren et al., 2018) in *C. rosea* during interspecific fungal interactions, and consequently, in biocontrol. Induced expression of fungal cell wall‐degrading enzymes including proteases and chitinases during interactions with other fungi (Lysøe et al., 2017; Nygren et al., 2018; Sun et al., 2015b), in combination with signs of diversifying selection (Iqbal, et al., 2018) or function in mycoparasitism (Sun et al., 2017; Tzelepis et al., 2015) is also reported for *C. rosea* and *C. chloroleuca*. Nygren et al. (2018) noted this striking overlap between gene family expansions (diversifying selection) and expression/function during interspecific fungal interactions and argued that it supports the view of mycoparasitism being an important factor for shaping the genome content in *C. rosea* and the here studied closely related other species.

We show that the expansion of GH43, AA3 and AA9 carbohydrate‐active enzyme gene families in *C. rosea* reported previously (Karlsson et al., 2015) is in fact a trait common to several closely related *Clonostachys* species. It is plausible to propose a link between the expansion of the AA9 lytic polysaccharide monooxygenases and the AA3 glucose–methanol–choline oxidoreductases, as the function of AA9 enzymes depends on exogenous electron donors (Vaaje‐Kolstad et al., 2010) that may be provided by AA3 cellobiose dehydrogenases (Vaaje‐Kolstad et al., 2010) and aryl alcohol oxidases and glucose 1‐oxidases (Kracher et al., 2016). The substrates targeted by the AA9 enzymes in *Clonostachys* spp. is currently not identified and requires further study. Several expanded carbohydrate‐active enzyme gene families in *Clonostachys* spp. are predicted to encode enzymes targeting plant cell wall polysaccharides, most notably xylan (CE3, GH3, GH16 and GH43) and rhamnose/pectin (CBM67 and GH78) substrates. *Clonostachys rosea* is also reported to have high numbers of PL1 pectin/pectate lyases (Atanasova et al., 2018; Karlsson et al., 2015). This may reflect the rhizosphere niche where *Clonostachys* spp. are commonly found and its phytophage capacity (Lübeck et al., 2002). *Clonostachys rosea* can also form very intimate relationships with plants through root surface colonization (Karlsson et al., 2015; Saraiva et al., 2015) and by penetrating epidermal cells (Yu & Sutton, 1997).

The origin of the *Clonostachys* genus in our study, and the considerable gene family expansions outlined above, is estimated to have happened 27 million years ago. We note that the origin of the three major *Trichoderma* sections/clades Harzianum/Virens, Longibrachiatum and Trichoderma are estimated to 20‐30 million years ago (Kubicek et al., 2019), where each section/clade is accompanied with extensive gene gains or losses. These coinciding events suggest certain environmental factors in the early Oligocene that may have promoted diversification of traits involved in mycoparasitism and consequently, of mycoparasitic species. This period was characterized by a mass extinction likely driven by cooler winters due to changing oceanographic and/or atmospheric circulation (Ivany et al., 2000). *Clonostachys* and *Trichoderma* appear to have evolved along separate evolutionary trajectories with regard to genome size; the relatively large genomes of *Clonostachys* spp. are in contrast to the smaller *Trichoderma* genomes. The large *Clonostachys* genomes may have counteracted the efficiency of genetic drift (Kelkar & Ochman, 2012), resulting in increased paralog numbers.

Regarding the wide occurrence of factors resulting in possible exploitation of *Clonostachys* spp. for biocontrol applications, we also acknowledge the fact that the species *C. rosea* stands out in many respects. Among the studied strains, we show that *C. rosea* strain IK726 has the largest genome, the highest number of genes and the highest number of gene family expansions. This may partly be an artefact due to the differences in sequencing technology, as PacBio sequencing of the *C. rosea* IK726 genome resulted in 12.4 Mbp additional sequence information and 3,240 additional genes (Broberg et al., 2018), compared with Illumina sequencing (Karlsson et al., 2015). However, the *C. solani* strain 1703 genome was also determined using PacBio technology and was the second smallest *Clonostachys* genome with the lowest number of genes. The failure to find any signs of active RIP mutations in the *C. rosea* genome may suggest that loss of this genome‐defence mechanism favoured sequence repeats (Hane et al., 2014) and resulted in lower selective constraints on gene family size. Gene family expansions uniquely found in *C. rosea* include the 1.B.12 Autotransporter‐1, the 1.C.113 Hly III and the 3.A.23 Type VI symbiosis/virulence secretory pathway families associated with virulence factors in biotic interactions (Baida & Kuzmin, 1996; Coulthurst, 2019; Drobnak et al., 2015), perhaps indicating a novel function in interspecific fungal interactions. The expansion of AA7 gluco‐ or chitooligosaccharide oxidases may contribute to polysaccharide degradation, either by oxidizing the substrate for subsequent hydrolysis or by production of hydrogen peroxide used by peroxidases (van Hellemond et al., 2006). Expansion of GH3 and GH16 glucanase/xylanase and GT1 glycosyltransferase gene families may indicate its phytophage potential, although it is difficult to speculate about their exact function without additional biochemical data.

However, the most conspicuous features of *C. rosea* are the expansions of the 2.A.1.3 drug:H^+^ antiporter‐2 MFS drug resistance transporter, the ABC‐B4 multidrug resistance transporter and the ABC‐C5 multidrug resistance‐associated transporter gene families. Several genes belonging to these families are also reported to be induced in *C. rosea* in response to fungal–fungal interactions or *Fusarium* metabolites, including *mfs464*, *mfs602*, *abcB17*, *abcB18*, *abcB19*, *abcB26* and *abcC8* (Demissie et al., 2018; Karlsson et al., 2015; Kosawang, et al., 2014; Nygren et al., 2018). The *abcB18*, *abcB20*, *abcC12* and *abcC14* genes are induced in response to bacterial metabolites (Kamou et al., 2016; Karlsson et al., 2015), while *mfs602* and *abcC8* are induced by exposure to xenobiotics (Nygren et al., 2018). Deletion of *mfs464* resulted in *C. rosea* mutants with increased in vitro antagonistic activity towards *F. graminearum* (Nygren et al., 2018). It is not far‐fetched to assume that this massive expansion of various drug efflux transporters provides *C. rosea* with efficient abilities to handle toxic secondary metabolite levels in its ecological niche leading to increased rhizosphere competence, phytophage potential and mycoparasitism.

In order to gain further support for this hypothesis, we can take a closer look at the ABC‐G1 pleiotropic drug resistance transporter gene family. Our gene family evolution and reconciliation analyses show that the expansion of this gene family coincided with the origin of *Clonostachys* subgenus *Bionectria*, resulting in high numbers of ABC‐G1 genes in the here tested, closely related *Clonostachys* species. Incongruence between species and gene phylogenies suggests an ongoing birth‐and‐death type of evolution although dominated by lineage‐specific gene loss. By investigating structural, regulatory and functional differences between *C. rosea* ABC‐G1 paralogs, we can test the hypothesis that the observed gene family expansion is driven by the selection for functional diversification. By comparing two paralogous ABC‐G1 groups, represented by the predicted *C. rosea* ABCG5 and ABCG6 proteins, we were able to identify several regions indicative of structural divergence between the groups. Although it is difficult to predict the structural effect from these differences, we note that one of these regions is located in the extracellular loop 6, involved in substrate transport in *Saccharomyces cerevisiae* Pdr5p and *Candida albicans* Cdr1p (Lamping et al., 2010). Furthermore, three regions are located in transmembrane helices possibly involved in substrate specificity (Lamping et al., 2010).

Different transcriptomic responses are induced in *C. rosea* during interactions with *B. cinerea* (representing alloparasitism) or *F. graminearum* (representing adelphoparasitism), suggesting that *C. rosea* developed different specific interaction mechanisms for attacking different fungal preys (Nygren et al., 2018). However, there are no obvious regulatory differences between *abcG5* and *abcG6*. *AbcG5* is reported to be induced by exposure to the antifungal *Fusarium* mycotoxin zearalenone, and the fungicides azoxystrobin, boscalid, iprodione and mefenoxam, but not during dual plate interactions with *B. cinerea* or *F. graminearum* (Dubey et al., 2014a), which is perfectly correlated with the transcriptional profile of *abcG6* in the current work. The phenotypic characterization of ABCG5 made by Dubey et al. (2014a) and ABCG6 in the current work shows that these paralogous transporters have overlapping, but distinct, functions. Neither *abcG5* nor *abcG6* gene deletion strains are affected in growth rate on CZ medium or on medium containing azoxystrobin, while both mutants have reduced growth rate on iprodione, compared with the wild‐type strain. In contrast with ∆*abcG6*, ∆*abcG5* strains have reduced growth rates on media containing mefenoxam and zearalenone (Dubey et al., 2014a). ABCG5 is also reported to protect against zearalenone and to be necessary for exploitation of *C. rosea* to protect barley seedlings against fusarium root rot (Dubey et al., 2014a), while ABCG6 is dispensable for these abilities. We conclude that functional divergence of ABC‐G1 pleiotropic drug resistance transporter paralogs and the observed selection for increased gene copy numbers contribute at least partially to fungal secondary metabolite and xenobiotic transport specificity in *Clonostachys*.

In summary, our comparative genomic analysis identified gene family expansions of PKS, NRPS, CYP, ABC and MFS transporters that emphasizes the role of biosynthesis of, and protection against, secondary metabolites for the evolution of several closely related species in subgenus *Bionectria*. The accumulating evidence that many members in these expanded gene families are specifically induced, or have a proven function, during interspecific microbial interactions, provides important clues for understanding the basis of the nutritional versatility that characterizes generalism behaviour in *Clonostachys*. Neutralization of antifungal compounds or antibiotics produced by other microorganisms, including defence molecules from other fungi, or defence molecules produced by plants may facilitate the ecological generalist lifestyle in *Clonostachys* subgenus *Bionectria*, which includes endophytism, rhizosphere competence, polyphage ability and mycoparasitism. The impressive catalogue of various drug efflux transporters in *Clonostachys* and the resulting tolerance against various xenobiotic substances may find applied uses when combining *C. rosea* with other BCAs with different mode of action (Kamou et al., 2016; Karlsson et al., 2015) or with low‐dose fungicide applications (Roberti et al., 2006). Finally, the availability of genome sequences of different species, as well as a population genomic dataset (Broberg et al., 2018), will be of great value for future studies of the evolution and biology of *Clonostachys*, ultimately facilitating knowledge‐based improvements in biocontrol efficacy in agricultural production.

## DATA ARCHIVING STATEMENT

The genome sequence data generated and analysed in this work are deposited in the European Nucleotide Archive (ENA) at EMBL under the accession number PRJEB32493.

## Supporting information

Fig S1

Fig S2

Fig S3

Fig S4

Fig S5

Fig S6

Fig S7

Table S1

Table S2

Table S3

Table S4

Table S5

Table S6

Supplementary Material

## Data Availability

The data that support the findings of this study are openly available in EMBL at the European Nucleotide Archive (ENA) [https://www.ebi.ac.uk/ena], reference number PRJEB32493.
