# Supplementary material for: Comparative genomics highlights the importance of drug efflux transporters during evolution of mycoparasitism in Clonostachys subgenus Bionectria (Fungi, Ascomycota, Hypocreales)
Source: Evol Appl. 2020 Sep 28;14(2):476–97. doi: 10.1111/eva.13134 (PMC7896725; doi:10.1111/eva.13134)
Supplement: Supplementary file 1 — Fig S1 [file EVA-14-476-s001.pdf]

**Supporting Information Figure S1**

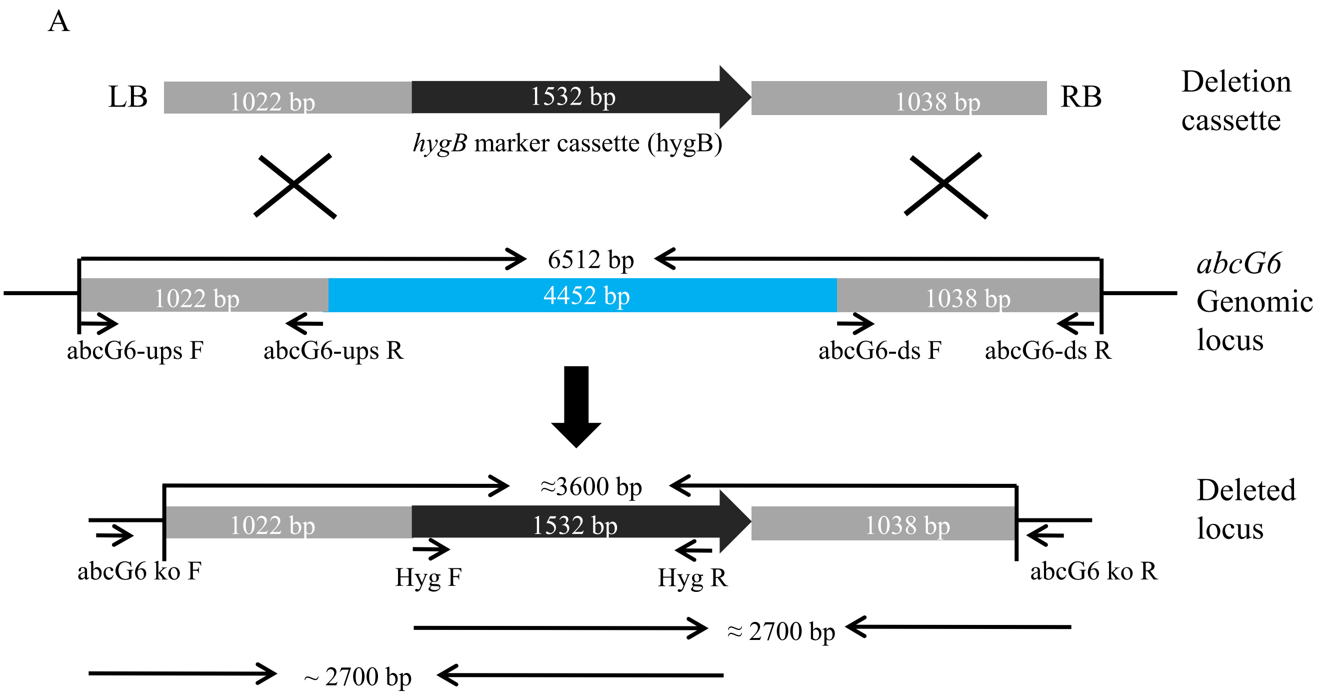


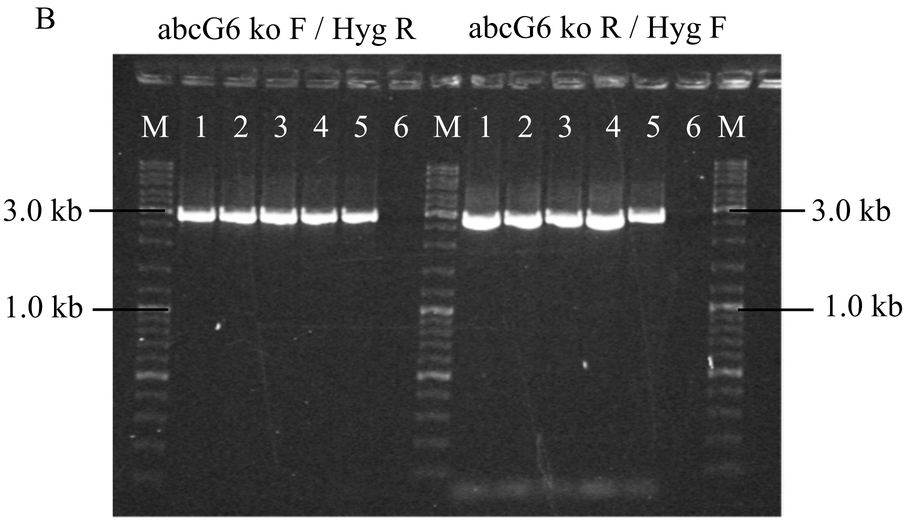


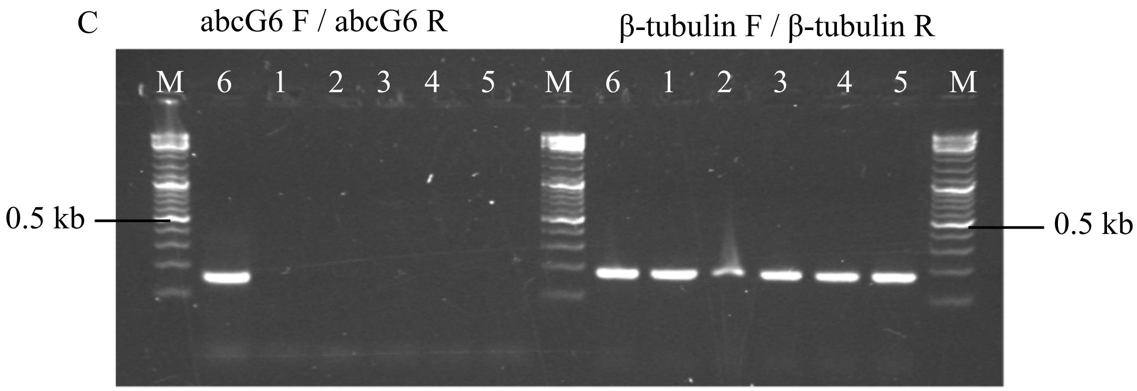

Schematic representation of deletion cassettes and characterization of mutant strains using PCR and RT-PCR.

**A**, Organisation of *abcG6* locus in wildtype (WT) and mutant strains of *C. rosea*. The *abcG6* gene was replaced by the hygB cassette by homologous recombination resulting in generation of Δ*abcG6* strains. The small arrow heads indicate the location of primers used to construct the deletion cassette and analysis of mutants using PCR. The large arrow heads indicate the size of amplified PCR products. Abbreviations: LB, left border; RB, right border.

**B**, PCR verification of Δ*abcG6* using primers located in the hygB cassette (Hyg F /Hyg R) in combination with primers located upstream and downstream from the deletion cassette (abcG6 ko F /abcG6 ko R). PCR products of ~2.7 kb using primers abcG6 ko F / Hyg R and abcG6 ko R / Hyg F, were expected from a correct gene replacement. M, gene ruler DNA ladder mix; 1-5, independent Δ*abcG6* mutants; 6, WT strain.

**C**, RT-PCR analysis of *abcG6* gene expression in WT and deletion strains using *abcG6* specific primers. M, gene ruler DNA ladder mix; 6, WT; 1-5, independent deletion strains. Primer combinations used for PCR and RT-PCR are given above the images.
