# Supplementary material for: Comparative genomics highlights the importance of drug efflux transporters during evolution of mycoparasitism in Clonostachys subgenus Bionectria (Fungi, Ascomycota, Hypocreales)
Source: Evol Appl. 2020 Sep 28;14(2):476–97. doi: 10.1111/eva.13134 (PMC7896725; doi:10.1111/eva.13134)
Supplement: Supplementary file 2 — Fig S2 [file EVA-14-476-s002.pdf]

Supporting Information Figure S2

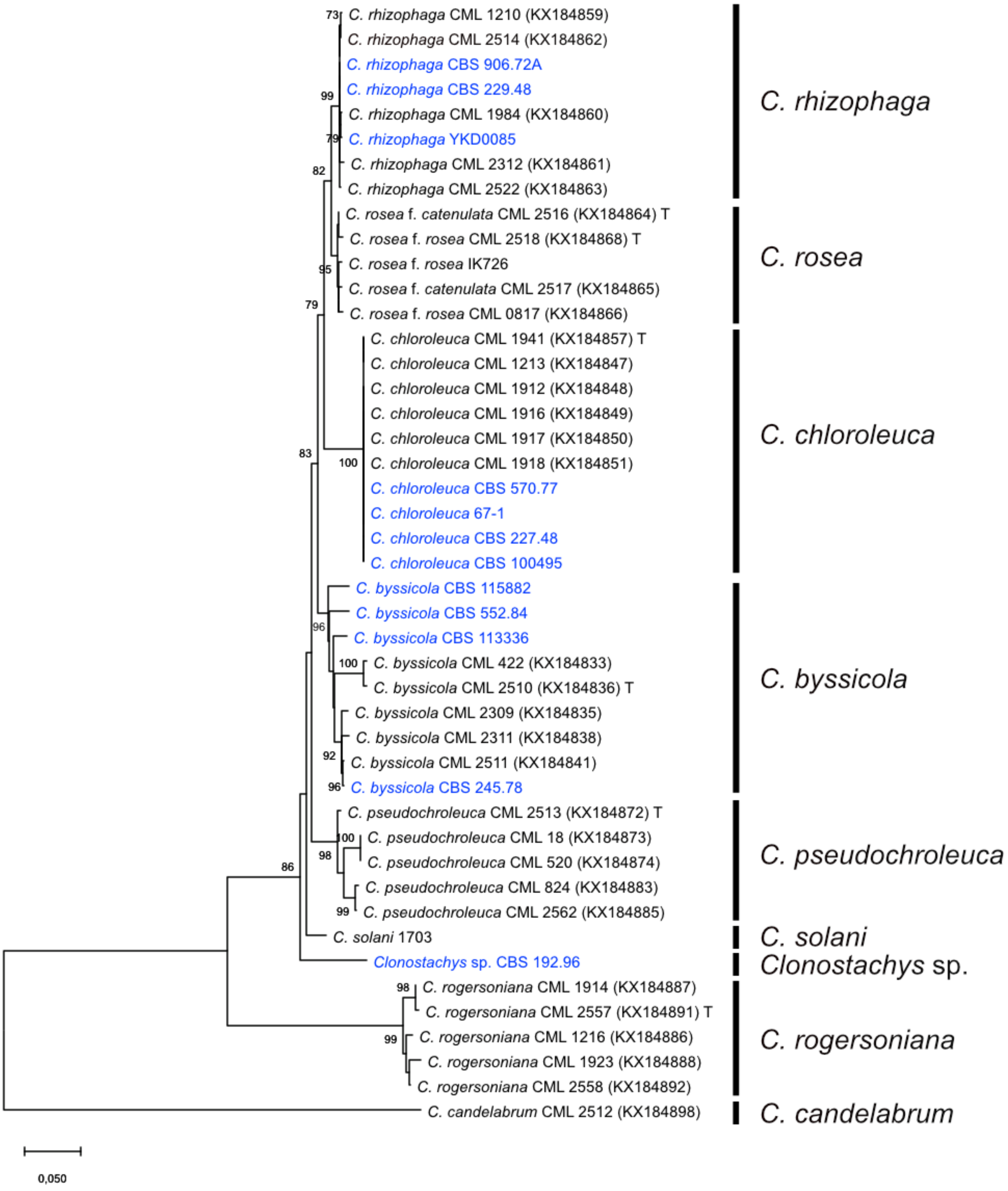

A. Maximum likelihood tree showing phylogenetic relatedness of *Clonostachys* subgenus *Bionectria* strains, based on partial ATP citrate lyase (*acl1*) gene sequences. The tree is rooted with *C. candelabrum* (*Clonostachys* subgenus *Epiphloea*). Bootstrap branch support values (≥ 70%) based on 1000 iterations are given. Sequence identifiers include species and strain ID, followed by sequence GenBank ID number in parenthesis (if available). The bar marker indicates average number of substitutions per site. Sequence identifiers in blue indicate new species name assignments based on the results from the current work. Letter T indicates ex-type strains.

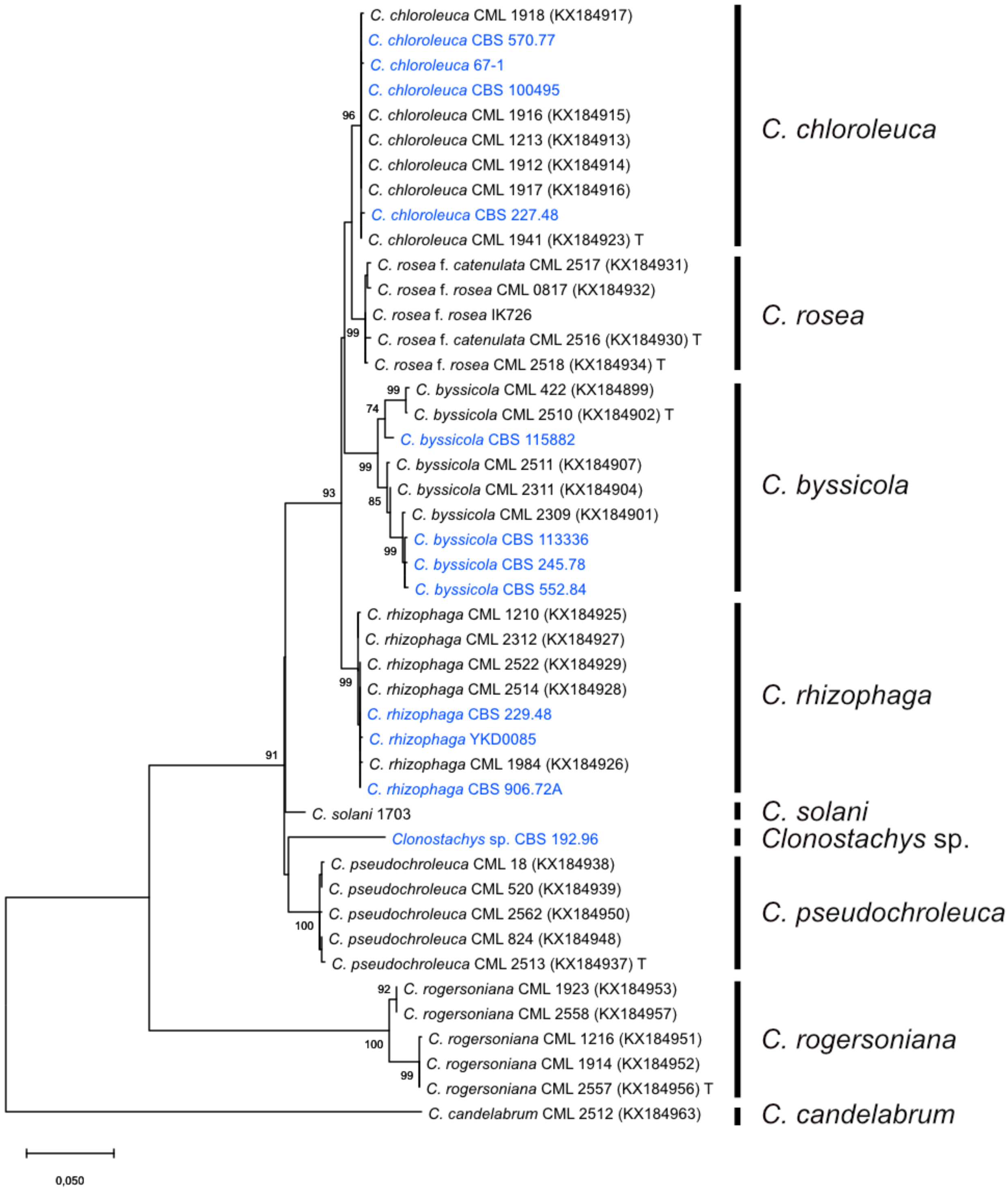

B. Maximum likelihood tree showing phylogenetic relatedness of *Clonostachys* subgenus *Bionectria* strains, based on partial RNA polymerase II large subunit (*rpb1*) gene sequences. The tree is rooted with *C. candelabrum* (*Clonostachys* subgenus *Epiphloea*). Bootstrap branch support values (≥ 70%) based on 1000 iterations are given. Sequence identifiers include species and strain ID, followed by sequence GenBank ID number in parenthesis (if available). The bar marker indicates average number of substitutions per site. Sequence identifiers in blue indicate new species name assignments based on the results from the current work. Letter T indicates ex-type strains.

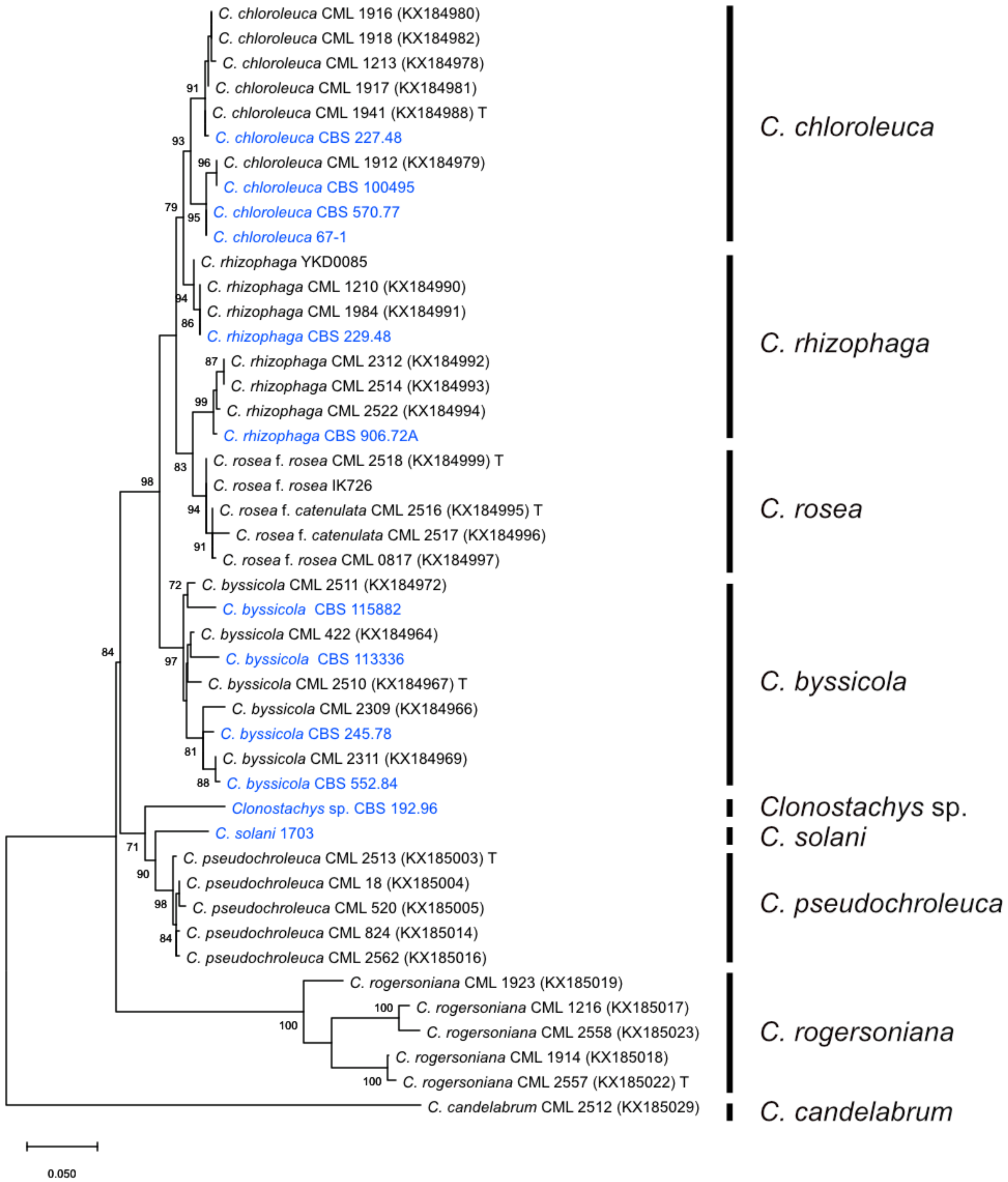

C. Maximum likelihood tree showing phylogenetic relatedness of *Clonostachys* subgenus *Bionectria* strains, based on partial translation elongation factor 1-α (*tef1*) gene sequences. The tree is rooted with *C. candelabrum* (*Clonostachys* subgenus *Epiphloea*). Bootstrap branch support values (≥ 70%) based on 1000 iterations are given. Sequence identifiers include species and strain ID, followed by sequence GenBank ID number in parenthesis (if available). The bar marker indicates average number of substitutions per site. Sequence identifiers in blue indicate new species name assignments based on the results from the current work. Letter T indicates ex-type strains.

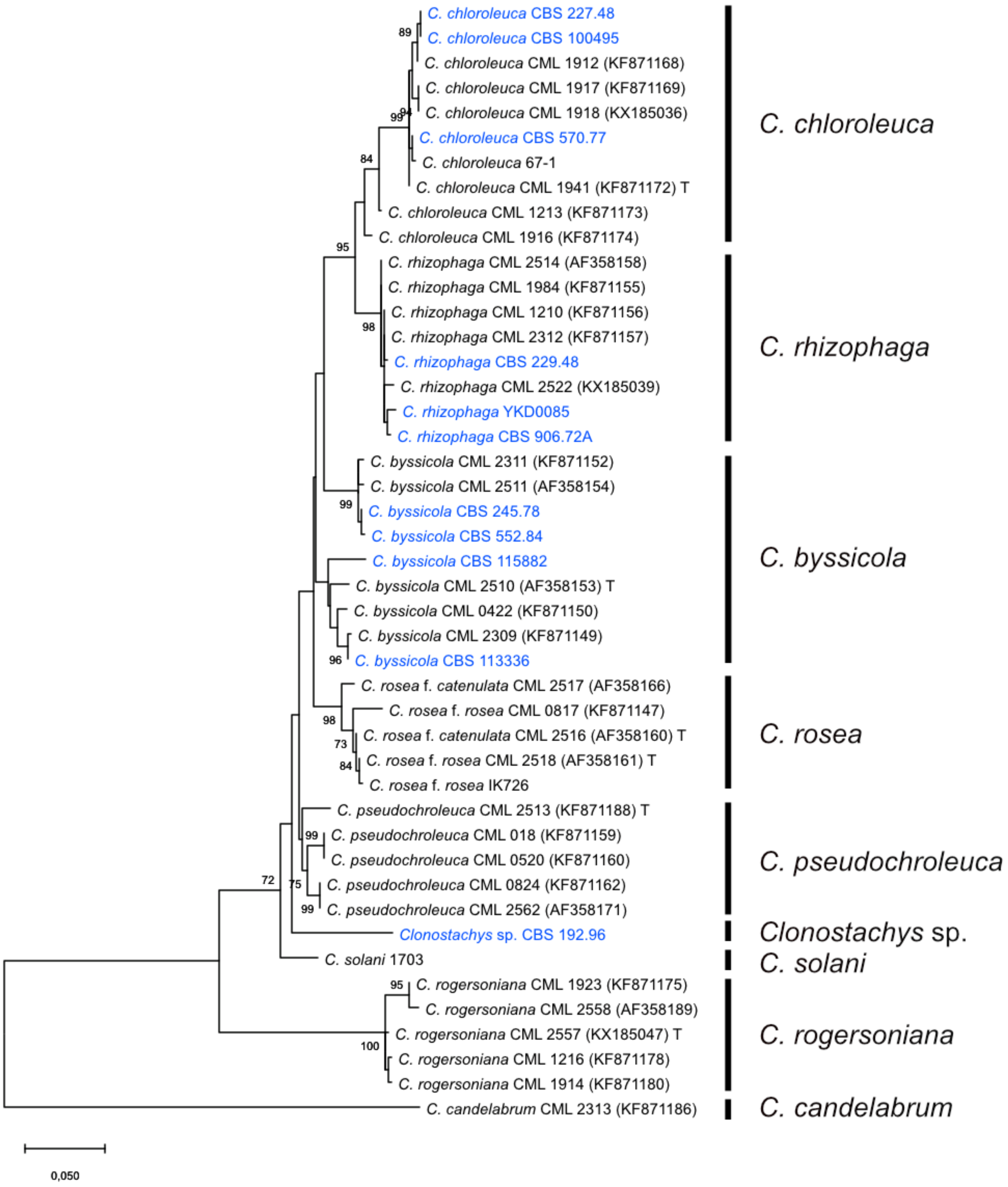

D. Maximum likelihood tree showing phylogenetic relatedness of *Clonostachys* subgenus *Bionectria* strains, based on partial β-tubulin (*tub*) gene sequences. The tree is rooted with *C. candelabrum* (*Clonostachys* subgenus *Epiphloea*). Bootstrap branch support values (≥ 70%) based on 1000 iterations are given. Sequence identifiers include species and strain ID, followed by sequence GenBank ID number in parenthesis (if available). The bar marker indicates average number of substitutions per site. Sequence identifiers in blue indicate new species name assignments based on the results from the current work. Letter T indicates ex-type strains.
