# Supplementary material for: Comparative genomics highlights the importance of drug efflux transporters during evolution of mycoparasitism in Clonostachys subgenus Bionectria (Fungi, Ascomycota, Hypocreales)
Source: Evol Appl. 2020 Sep 28;14(2):476–97. doi: 10.1111/eva.13134 (PMC7896725; doi:10.1111/eva.13134)
Supplement: Supplementary file 3 — Fig S3 [file EVA-14-476-s003.pdf]

Supporting Information Figure S3

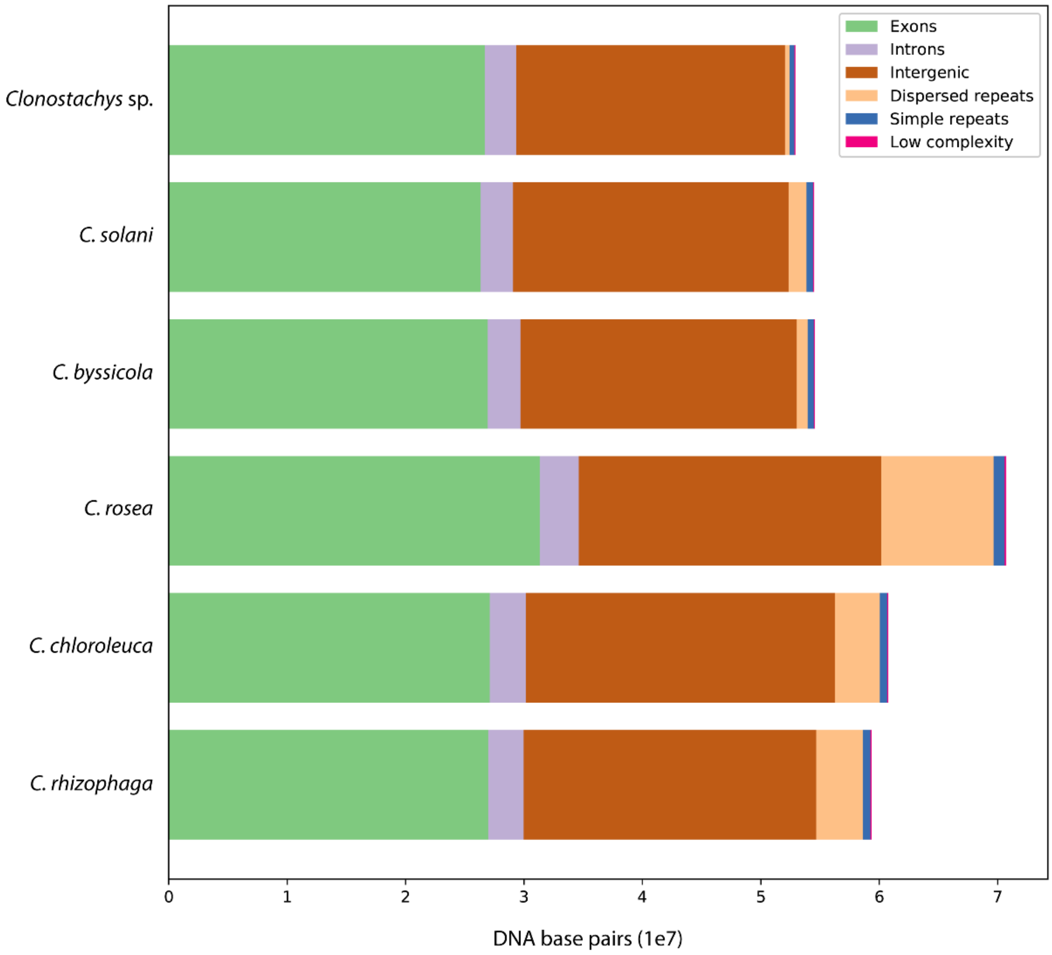

Supporting Information Figure S3. Distribution of genome content in *Clonostachys*. Genomes were annotated using a MAKER-based pipeline. The annotations from MAKER was used to classify the genome sequences into functional categories, including exons, introns, intergenic regions, dispersed repeats, simple repeats and low complexity regions. Strains included were *C. solani* 1703, *C. byssicola* CBS 245.78, *C. rhizophaga* CBS 906.72A, *C. chloroleuca* CBS 570.77, *Clonostachys* sp. CBS 192.96 and *C. rosea* IK726.
