# Supplementary figures and images for: Comparative genomics highlights the importance of drug efflux transporters during evolution of mycoparasitism in Clonostachys subgenus Bionectria (Fungi, Ascomycota, Hypocreales)
Source: Evol Appl. 2020 Sep 28;14(2):476–97. doi: 10.1111/eva.13134 (PMC7896725; doi:10.1111/eva.13134)

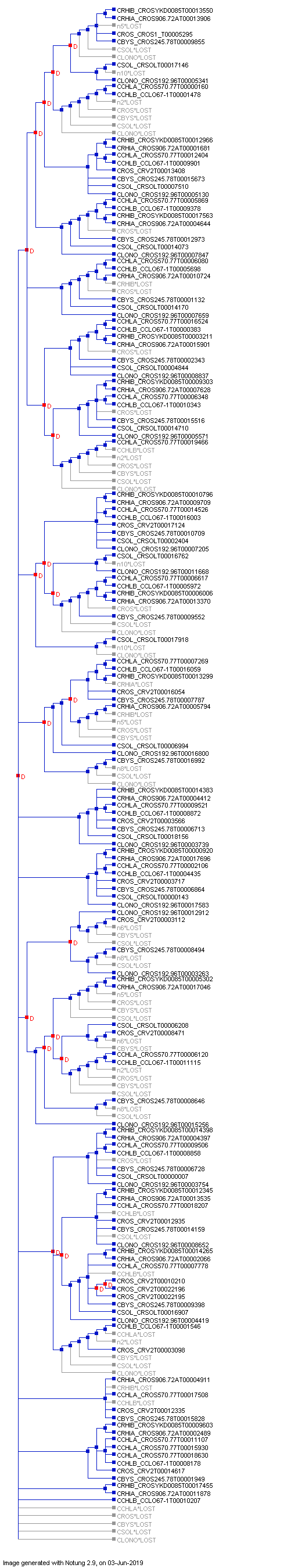

Supplement: Supplementary file 5 — Fig S5 [file EVA-14-476-s005.png]
