# Supplementary material for: Comparative genomics highlights the importance of drug efflux transporters during evolution of mycoparasitism in Clonostachys subgenus Bionectria (Fungi, Ascomycota, Hypocreales)
Source: Evol Appl. 2020 Sep 28;14(2):476–97. doi: 10.1111/eva.13134 (PMC7896725; doi:10.1111/eva.13134)
Supplement: Supplementary file 7 — Fig S7 [file EVA-14-476-s007.pdf]

Supporting Information Figure S7

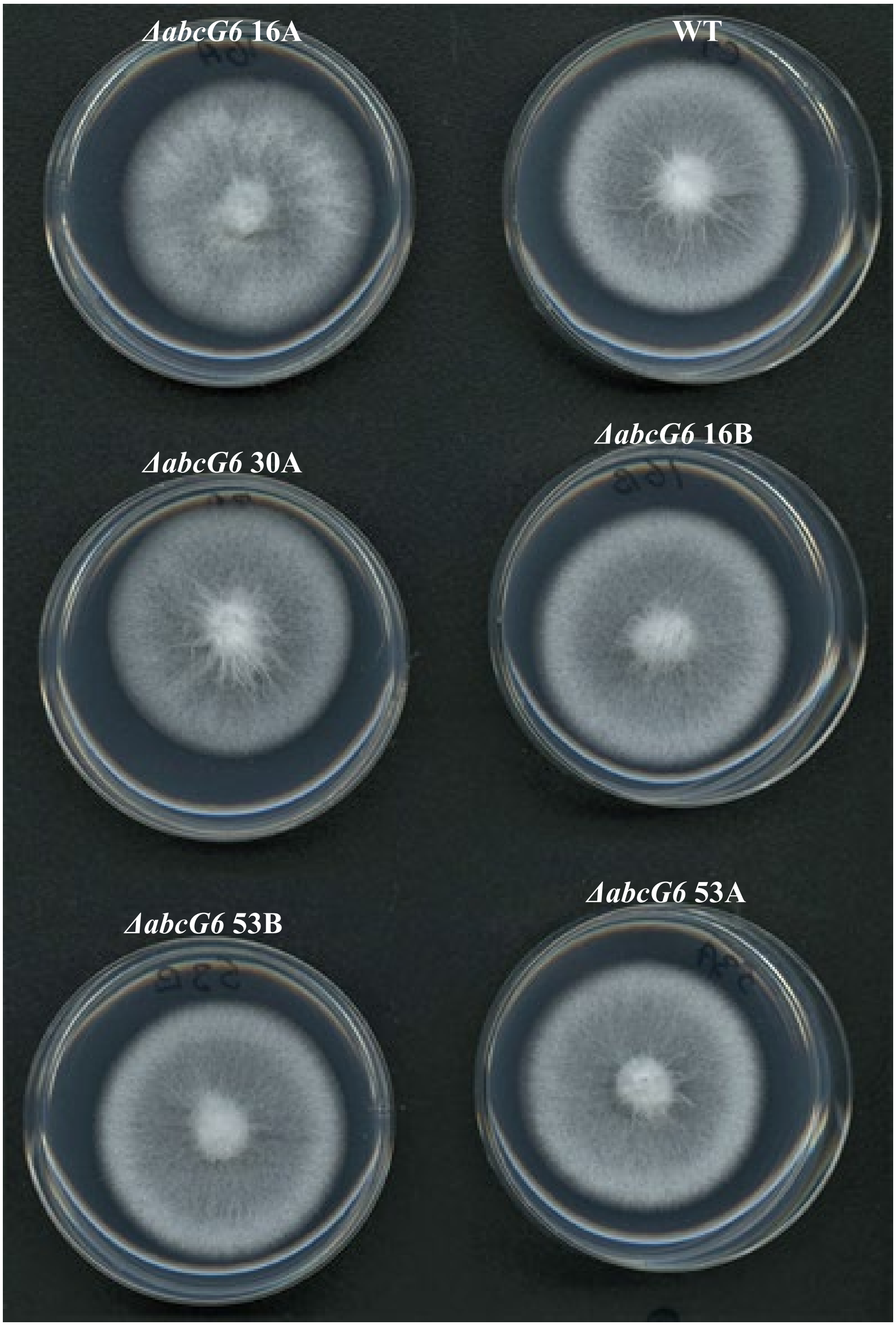

Supporting Information Figure S7: Colony morphology of *Clonostachys rosea* wildtype (WT) and Δ*abcG6* strains. *C. rosea* WT and *abcG6* deletion strains (16A, 16B, 30A, 53A and 53B) were inoculated on solid CZ medium and incubated at 25 °C for 5 days. The experiment was performed in three biological replicates and photographs of representative plates were taken by scanning the plates using Epson Perfection V700 Photo (Epson, Suwa, Japan).
