# Supplementary material for: Comparative genomics highlights the importance of drug efflux transporters during evolution of mycoparasitism in Clonostachys subgenus Bionectria (Fungi, Ascomycota, Hypocreales)
Source: Evol Appl. 2020 Sep 28;14(2):476–97. doi: 10.1111/eva.13134 (PMC7896725; doi:10.1111/eva.13134)
Supplement: Supplementary file 8 — Table S1 [file EVA-14-476-s008.pdf]

Supporting Information Table S1.

Taxonomic authorities of species used in the current study.

Species	Authors	Mycobank ID	Mycobank link
Botrytis cinerea	Persoon	#217312	http://www.mycobank.org/MB/217312
Clonostachys byssicola	Schroers	#485119	http://www.mycobank.org/MB/485119
Clonostachys chloroleuca	G.M. Moreira, L.M. Abreu, L.H. Pfenning & H.J. Schroers	#816994	http://www.mycobank.org/MB/816994
Clonostachys rhizophaga	Schroers	#485120	http://www.mycobank.org/MB/485120
Clonostachys rosea	(Link) Schroers, Samuels, K.A. Seifert & W. Gams	#461067	http://www.mycobank.org/MB/461067
Clonostachys solani	(Harting) Schroers & W. Gams	#456098	http://www.mycobank.org/MB/456098
Fusarium graminearum	Schwabe	#200256	http://www.mycobank.org/MB/200256
