# Supplementary material for: Comparative genomics highlights the importance of drug efflux transporters during evolution of mycoparasitism in Clonostachys subgenus Bionectria (Fungi, Ascomycota, Hypocreales)
Source: Evol Appl. 2020 Sep 28;14(2):476–97. doi: 10.1111/eva.13134 (PMC7896725; doi:10.1111/eva.13134)
Supplement: Supplementary file 9 — Table S2 [file EVA-14-476-s009.pdf]

Supporting Information Table S1

Gene Family Prediction and Analysis

Predicted proteomes of all strains were all collected into one directory, which was analyzed by Orthofinder v1.1.8 using the following command:

orthofinder -f /path/ProteinDirectory -t 30

The resulting output from Orthofinder was prepared and used in Kinfin.

./kinfin -g OrthofinderOuput/OrthologousGroups.txt -c OrthofinderOuput/config.txt -s OrthofinderOuput/SequenceIDs.txt -p OrthofinderOuput/SpeciesIDs.txt

As well as the following sub scripts were used:

plot_cluster_size_distribution.py
get_count_matrix.py
generate_network.py
get_protein_ids_from_cluster.py
ips_to_table.py
functional_annotation_of_clusters.py

BLAST (ver 2.2.27) analysis was performed using the following parameters:

blastp -evalue 1e-5 -outfmt "6 qseqid sseqid pident length mismatch gapopen qstart qend sstart send evalue bitscore sseq"

HMMER 3.0 analysis was performed with the following parameters.

hmmscan --domtblout

The results were subsequently filtered as described.

CAFE analysis was performed using the following CAFE script

```
#!/proj/mykopat-c-ros/software/cafehahnlab-code/cafe/cafe

date

#specify data file, p-value threshold, # of threads to use, and log file

load -i ProteinCategories.txt -t 30 -l logfile.txt -p 0.05



#the phylogenetic tree structure with branch lengths

tree	((((((((906-72A:0.20,C.ros.YKD0085:0.20):0.9,(C.chloroleuca67-1:0.02,570-77:0.02):1.08):0.10,C.ros.v2:1.20):0.30,245-78:1.50):1.00,C.solani:2.50):1.80,192-96:4.30):22.70,(F.solani:18.00,(F.gram:11.00,F.verticillioides:11.00):7.00):9.00):4.00,(T.atroviride:16.00,(Trichoderma.reesei:12.00,T.virens:12.00):4.00):15.00):3.00,N.crassa:34)

#lambda1

lambda -s -t ((((((((1,1)1,(1,1)1)1,1)1,1)1,1)1,1)1,(2,(2,2)2)2)1,(3,(3,3)3)3)1,4)

#report

report TCDBE40resultsReport
```
