# Supplementary material for: Comparative genomics highlights the importance of drug efflux transporters during evolution of mycoparasitism in Clonostachys subgenus Bionectria (Fungi, Ascomycota, Hypocreales)
Source: Evol Appl. 2020 Sep 28;14(2):476–97. doi: 10.1111/eva.13134 (PMC7896725; doi:10.1111/eva.13134)
Supplement: Supplementary file 14 — Supplementary Material [file EVA-14-476-s014.docx]

**Comparative genomics highlights the importance of drug efflux transporters during evolution of mycoparasitism in *Clonostachys* subgenus *Bionectria* (Fungi, Ascomycota, Hypocreales)**

**Supporting Information Figure legends**

Supporting Information Figure S1.

Schematic representation of deletion cassettes and characterization of mutant strains using PCR and RT-PCR.

A, Organisation of *abcG6* locus in wildtype (WT) and mutant strains of *C. rosea*. The *abcG6* gene was replaced by the hygB cassette by homologous recombination resulting in generation of Δ*abcG6* strains. The small arrow heads indicate the location of primers used to construct the deletion cassette and analysis of mutants using PCR. The large arrow heads indicate the size of amplified PCR products. Abbreviations: LB, left border; RB, right border.

B, PCR verification of Δ*abcG6* using primers located in the hygB cassette (Hyg F /Hyg R) in combination with primers located upstream and downstream from the deletion cassette (abcG6 ko F /abcG6 ko R). PCR products of ~2.7 kb using primers abcG6 ko F / Hyg R and abcG6 ko R / Hyg F, were expected from a correct gene replacement. M, gene ruler DNA ladder mix; 1-5, independent Δ*abcG6* mutants; 6, WT strain.

C, RT-PCR analysis of *abcG6* gene expression in WT and deletion strains using *abcG6* specific primers. M, gene ruler DNA ladder mix; 6, WT; 1-5, independent deletion strains.

Primer combinations used for PCR and RT-PCR are given above the images.

Supporting Information Figure S2.

A. Maximum likelihood tree showing phylogenetic relatedness of *Clonostachys* subgenus *Bionectria* strains, based on partial ATP citrate lyase (*acl1*) gene sequences. The tree is rooted with *C. candelabrum* (*Clonostachys* subgenus *Epiphloea*). Bootstrap branch support values (≥ 70%) based on 1000 iterations are given. Sequence identifiers include species and strain ID, followed by sequence GenBank ID number in parenthesis (if available). The bar marker indicates average number of substitutions per site. Sequence identifiers in blue indicate new species name assignments based on the results from the current work. Letter T indicates ex-type strains.

B. Maximum likelihood tree showing phylogenetic relatedness of *Clonostachys* subgenus *Bionectria* strains, based on partial RNA polymerase II large subunit (*rpb1*) gene sequences. The tree is rooted with *C. candelabrum* (*Clonostachys* subgenus *Epiphloea*). Bootstrap branch support values (≥ 70%) based on 1000 iterations are given. Sequence identifiers include species and strain ID, followed by sequence GenBank ID number in parenthesis (if available). The bar marker indicates average number of substitutions per site. Sequence identifiers in blue indicate new species name assignments based on the results from the current work. Letter T indicates ex-type strains.

C. Maximum likelihood tree showing phylogenetic relatedness of *Clonostachys* subgenus *Bionectria* strains, based on partial translation elongation factor 1-α (*tef1*) gene sequences. The tree is rooted with *C. candelabrum* (*Clonostachys* subgenus *Epiphloea*). Bootstrap branch support values (≥ 70%) based on 1000 iterations are given. Sequence identifiers include species and strain ID, followed by sequence GenBank ID number in parenthesis (if available). The bar marker indicates average number of substitutions per site. Sequence identifiers in blue indicate new species name assignments based on the results from the current work. Letter T indicates ex-type strains.

D. Maximum likelihood tree showing phylogenetic relatedness of *Clonostachys* subgenus *Bionectria* strains, based on partial β-tubulin (*tub*) gene sequences. The tree is rooted with *C. candelabrum* (*Clonostachys* subgenus *Epiphloea*). Bootstrap branch support values (≥ 70%) based on 1000 iterations are given. Sequence identifiers include species and strain ID, followed by sequence GenBank ID number in parenthesis (if available). The bar marker indicates average number of substitutions per site. Sequence identifiers in blue indicate new species name assignments based on the results from the current work. Letter T indicates ex-type strains.

Supporting Information Figure S3.

Distribution of genome content in *Clonostachys*. Genomes were annotated using a MAKER-based pipeline. The annotations from MAKER was used to classify the genome sequences into functional categories, including exons, introns, intergenic regions, dispersed repeats, simple repeats and low complexity regions. Strains included were *C. solani* 1703, *C. byssicola* CBS 245.78, *C. rhizophaga* CBS 906.72A, *C. chloroleuca* CBS 570.77, *Clonostachys* sp. CBS 192.96 and *C. rosea* IK726.

Supporting Information Figure S4.

Newick tree file describing the phylogenetic relationships of *Clonostachys* spp. ABC-G1 pleiotropic drug resistance transporters. Predicted ABC transporter protein sequences were aligned with MUSCLE (Edgar, 2004) and phylogenetic analysis was performed using maximum likelihood methods implemented in MEGA ver. 6 (Tamura, et al. 2013). The LG (Le & Gascuel, 2008) +G+F amino acid substitution model was used along with all sites. Statistical support for branches was assessed by 500 bootstrap resampling. Locus tags: CROS245.78 = *C. byssicola* CBS 245.78, CROS570.77 = *C. chloroleuca* CBS 570.77, CCLO67-1 = *C. chloroleuca* 67-1, CROS906.72A = *C. rhizophaga* CBS 906.72A, CROSYKD0085 = *C. rhizophaga* YKD0085, CRV2/CROS1 = *C. rosea* IK726, CRSOL = *C. solani* 1703, CROS192.96 = *Clonostachys* sp. CBS 192.96.

Supporting Information Figure S5.

Reconciliation of *Clonostachys* spp. ABC-G1 gene tree with the species tree by NOTUNG. Nodes marked in red and the letter D indicates a gene duplication event, while terminal branches marked in grey indicates a gene loss. Abbreviations: CBYS = *C. byssicola* CBS 245.78, CCHLA = *C. chloroleuca* CBS 570.77, CCHLB = *C. chloroleuca* 67-1, CRHIA = *C. rhizophaga* CBS 906.72A, CRHIB = *C. rhizophaga* YKD0085, CROS = *C. rosea* IK726, CSOL = *C. solani* 1703, CLONO = *Clonostachys* sp. CBS 192.96. Locus tags as described in Supporting Information Figure S4.

Supporting Information Figure S6.

Predicted ABCG5 and ABCG6 ABC transporter protein sequences were aligned with MUSCLE (Edgar, 2004). Locus tags as described in Supplementary figure 2.

Supporting Information Figure S7.

Colony morphology of *Clonostachys* *rosea* wildtype (WT) and *∆abcG6* strains. *C*. *rosea* WT and *abcG6* deletion strains (16A, 16B, 30A, 53A and 53B) were inoculated on solid CZ medium and incubated at 25°C for 5 days. The experiment was performed in three biological replicates and photographs of representative plates were taken by scanning the plates using Epson Perfection V700 Photo (Epson, Suwa, Japan).
